# Supplementary material for: Exploring the Reported Strengths and Limitations of Aboriginal and Torres Strait Islander Health Research: A Narrative Review of Intervention Studies
Source: Int J Environ Res Public Health. 2023 Feb 23;20(5):3993. doi: 10.3390/ijerph20053993 (PMC10001772; doi:10.3390/ijerph20053993)
Supplement: Supplementary file 1 [file ijerph-20-03993-s001.zip › ijerph-2133517-supplementary.pdf]

Table S1. Summary of the strengths and limitations reported in the studies (n = 240).

Themes	Evaluations *n* (%)	Implementations *n* (%)	Pilot interventions *n* (%)	Trials *n* (%)
**Strengths**				
Community engagement and partnerships (*n* = 64)	25 (22.73%)	20 (45.45%)	9 (30.00%)	10 (17.86%)
Sample qualities (*n* = 32)	11 (10.00%)	4 (9.09%)	4 (13.33%)	13 (23.21%)
Aboriginal and Torres Strait Islander Research Team (*n* = 21)	8 (7.27%)	5 (11.36%)	5 (16.67%)	3 (5.36%)
Culturally appropriate and safe research practice (*n* = 17)	6 (5.45%)	7 (15.91%)	5 (16.67%)	0 (0.00%)
Capacity building efforts (*n* = 12)	4 (3.64%)	4 (9.09%)	4 (13.33%)	1 (1.79%)
Providing resources or reducing costs for services and communities (*n* = 9)	3 (2.73%)	5 (11.36%)	1 (3.33%)	0 (0.00%)
Understanding local culture and context (*n* = 7)	4 (3.64%)	1 (2.27%)	2 (6.67%)	0 (0.00%)
Appropriate timelines for completion (*n* = 2)	1 (0.91%)	1 (2.27%)	0 (0.00%)	0 (0.00%)
**Limitations**				
Difficulties achieving the target sample size (*n* = 119)	46 (41.82%)	18 (40.91%)	22 (73.33%)	33 (58.93%)
Inadequate time to conduct research (*n* = 23)	10 (9/09%)	5 (11.36%)	6 (20.00%)	2 (3.57%)
Funding, costs, and resources (*n* = 15)	8 (7.27%)	2 (4.55%)	4 (13.33%)	1 (1.79%)
Limited capacity of health workers and services (*n* = 12)	4 (3.64%)	1 (2.27%)	6 (20.00%)	1 (1.79%)
Lack of community involvement (*n* = 3)	1 (0.91%)	1 (2.27%)	0 (0.00%)	1 (1.79%)
Communication issues between communities and researchers (*n* = 3)	1 (0.91%)	0 (0.00%)	2 (6.67%)	0 (0.00%)

Note. Evaluations (n =110), implementations (n = 44), pilot interventions (n = 30), and trials (n = 56). The cells represent the number and percentages of studies within the theme by publication classification.

Table S2. Detailed characteristics for each intervention article, 2008–2020.

Study	Publication classification	Journal	State	Remoteness	Barriers	Enablers
Abbott 2013 [1]	Implementation	BMC Public Health	NSW	Urban		
Adams 2012 [2]	Evaluation	Health Soc Care Community	VIC	Urban		
Andrews 2009 [3]	Implementation	PLoS Negl Trop Dis	NT	Remote		Community engagement and partnerships
Anikeeva 2012 [4]	Evaluation	Aust Fam Physician	Other	Not stated		
Anstey 2011 [5]	Evaluation	Aust N Z J Public Health	National	National		Community engagement and partnerships, Aboriginal and Torres Strait Islander research team
Armstrong 2020 [6]	Implementation	PLoS One	NSW, VIC, QLD	Rural, Remote, Urban	Difficulties achieving the target sample size, inadequate time to conduct research	
Arrow 2021 [7]*	Trial	JDR Clin Trans Res	WA	Remote		Community engagement and partnerships, Aboriginal and Torres Strait Islander research team
Askew 2019 [8]	Evaluation	BMC Public Health	QLD	Urban	Difficulties achieving the target sample size, inadequate time to conduct research, funding/cost/resources	Community engagement and partnerships, Aboriginal and Torres Strait Islander research team
Askew 2016 [9]	Evaluation	BMC Health Serv Res	QLD	Urban		
Atkinson 2014 [10]	Evaluation	Aust J Prim Health	NT	Remote		Community engagement and partnerships
Bailie 2015 [11]	Evaluation	BMJ Open	National	Regional, Rural, Remote		Community engagement and partnerships, sample qualities, providing resources or reducing costs for services and communities, culturally appropriate and safe research practice
Bailie 2011 [12]	Evaluation	J Epidemiol Community Health	NT	Remote		
Barrett 2015 [13]	Implementation	Aust J Rural Health	NSW	Rural		
Bar-Zeev 2019 [14]	Pilot intervention	BMJ Open	NSW, SA, QLD	Regional, Urban	Difficulties achieving the target sample size	Sample qualities
Battersby 2018 [15]	Implementation	Aust J Rural Health	National	Not stated		Community engagement and partnerships, Sample qualities

Bennett-Levy 2017 [16]	Implementation	J Med Internet Res	NSW	Urban		
Berends 2018 [17]	Evaluation	Aust J Rural Health	VIC	Not stated		
Bernardes 2018 [18]	Pilot intervention	Eur J Cancer Care	QLD	Not stated	Difficulties achieving the target sample size	
Bertilone 2015 [19]	Implementation	Med J Aust	WA	Urban		
Bierbaum 2017 [20]	Evaluation	Rural Remote Health	SA	Regional, Urban	Difficulties achieving the target sample size	Culturally appropriate and safe research practice
Biggs 2016 [21]	Evaluation	Sex Health	NSW	Urban		Community engagement and partnerships
Binks 2015 [22]	Trial	Vaccine	NT	Remote, Urban	Difficulties achieving the target sample size	Sample qualities
Binks 2018 [23]	Trial	Pneumonia	NT	Remote, Urban	Difficulties achieving the target sample size	
Bird 2016 [24]	Evaluation	Health Justice	QLD	Regional, Rural, Remote		
Black 2013a [25]	Evaluation	Med J Aust	NSW	Not stated		Providing resources or reducing costs for services and communities
Black 2013b [26]	Evaluation	Br J Nutr	NSW	Not stated		
Black 2014 [27]	Evaluation	Aust N Z J Public Health	NSW	Not stated		
Blignault 2016 [28]	Evaluation	Aust N Z J Public Health	SA, WA, NT, QLD	Regional, Remote		Community engagement and partnerships
Borg 2018 [29]	Trial	Vaccine	VIC	Not stated	Lack of community involvement	
Bowen 2016 [30]	Trial	Epidemiol Infect	NT	Remote	Difficulties achieving the target sample size	
Bowen 2014 [31]	Trial	BMC Infect Dis	NT	Remote		Sample qualities
Boyle 2017 [32]	Evaluation	Aust J Rural Health	QLD	Not stated		
Bradley 2020 [33]	Implementation	BMC Health Serv Res	NSW, SA, WA, NT, ACT	Regional, Remote, Urban	Difficulties achieving the target sample size, inadequate time to conduct research	Community engagement and partnerships, sample qualities
Bradshaw 2015 [34]	Implementation	Aust J Rural Health	WA	Not stated		Community engagement and partnerships, providing resources or reducing costs for

						services and communities, understanding local culture and context
Brimblecombe 2017a [35]	Evaluation	SSM Popul Health	NT, QLD	Remote	Funding/cost/resources, lack of community involvement	
Brimblecombe 2018 [36]	Trial	Br J Nutr	NT	Remote	Difficulties achieving the target sample size, funding/cost/resources	Community engagement and partnerships, sample qualities, capacity building efforts
Brimblecombe 2017b [37]	Trial	Lancet Public Health	NT	Remote	Difficulties achieving the target sample size	
Brown 2019 [38]	Evaluation	Int J Environ Res Public Health	QLD	Remote	Difficulties achieving the target sample size, inadequate time to conduct research, limited capacity of health workers and services	Community engagement and partnerships
Browne 2019a [39]	Evaluation	Public Health Nutr	National	National	Difficulties achieving the target sample size, inadequate time to conduct research	
Browne 2014 [40]	Evaluation	Aust J Prim Health	VIC	Not stated	Difficulties achieving the target sample size	Community engagement and partnerships, capacity building efforts
Browne 2019b [41]	Evaluation	Health Promot J Austr	National	Not stated	Difficulties achieving the target sample size	
Burgess 2008 [42]	Implementation	Aust Fam Physician	NT	Remote	Difficulties achieving the target sample size	
Cameron 2014 [43]	Implementation	Int J Audiol	NSW	Urban		
Campbell 2014 [44]	Evaluation	Aust J Prim Health	NSW	Not stated		
Campbell 2018 [45]	Evaluation	BMC Pregnancy Childbirth	QLD	Remote	Difficulties achieving the target sample size, funding/cost/resources	
Canuto 2013 [46]	Evaluation	Int J Equity Health	SA	Urban	Difficulties achieving the target sample size	
Canuto 2012 [47]	Trial	BMC Public Health	SA	Urban	Difficulties achieving the target sample size	
Cargo 2011 [48]	Evaluation	BMC Public Health	NT	Remote		Community engagement and partnerships
Cartwright 2018 [49]	Evaluation	Int J Environ Res Public Health	QLD	Not stated		Culturally appropriate and safe research practice
Cashman 2016 [50]	Evaluation	BMC Public Health	NSW	Urban		
Causer 2015 [51]	Evaluation	Sex Health	Other	Remote	Difficulties achieving the target sample size	
Hulme 2018 [52]	Evaluation	Rural Remote Health	VIC	Regional		

Chenhall 2008 [53]	Evaluation	Anthropol Med	Other	Rural		
Christou 2013 [54]	Evaluation	Contemp Nurse	WA	Not stated	Difficulties achieving the target sample size, limited capacity of health workers and services	
Ciccone 2019 [55]	Pilot intervention	Int J Speech Lang Pathol	WA	Not stated	Difficulties achieving the target sample size, limited capacity of health workers and services	Capacity building efforts, culturally appropriate and safe research practice
Clapham 2018 [56]	Evaluation	Health Promot J Austr	NSW	Urban	Limited capacity of health workers and services	
Clark 2015 [57]	Pilot intervention	Rural Remote Health	QLD	Not stated	Difficulties achieving the target sample size	Aboriginal and Torres Strait Islander research team, capacity building efforts, understanding local culture and context
Clifford 2011 [58]	Implementation	Drug Alcohol Rev	Other	Rural, Urban	Difficulties achieving the target sample size	Community engagement and partnerships
Clifford 2013 [59]	Implementation	Drug Alcohol Rev	NSW	Rural, Urban	Difficulties achieving the target sample size, inadequate time to conduct research	
Coffin 2019 [60]	Implementation	Front Public Health	WA	Not stated	Inadequate time to conduct research	Aboriginal and Torres Strait Islander research team, sample qualities
Conigrave 2012 [61]	Pilot intervention	Health Promot J Austr	NSW	Urban	Difficulties achieving the target sample size, inadequate time to conduct research, funding/costs/resources	Community engagement and partnerships
Cooper 2012 [62]	Pilot intervention	J Paediatr Child Health	NT	Remote	Difficulties achieving the target sample size	
Cresp 2016 [63]	Evaluation	Med J Aust	WA	Remote, Urban	Difficulties achieving the target sample size	
Crouch 2011 [64]	Implementation	Australas Psychiatry	QLD	Remote	Funding/costs/resources	Community engagement and partnerships
Cuesta-Briand 2015 [65]	Evaluation	Int J Equity Health	Other	Not stated		
d'Abbs 2008 [66]	Evaluation	Aust J Rural Health	QLD	Remote		
Davey 2014 [67]	Evaluation	BMC Health Serv Res	TAS	Urban	Difficulties achieving the target sample size	
Davies 2020 [68]	Implementation	Aust J Rural Health	NSW	Rural	Difficulties achieving the target sample size	Culturally appropriate and safe research practice
Daws 2014 [69]	Implementation	Aust Health Rev	VIC	Urban	Difficulties achieving the target sample size	Community engagement and partnerships, providing resources or reducing costs for services and communities

DiGiacomo 2010 [70]	Evaluation	Qual Prim Care	Other	Not stated		Community engagement and partnerships, culturally appropriate and safe research practice
Dimer 2013 [71]	Implementation	Aust Health Rev	WA	Not stated		Community engagement and partnerships, culturally appropriate and safe research practice
Dimitropoulos 2019 [72]	Implementation	Health Promot J Austr	NSW	Rural	Difficulties achieving the target sample size	Community engagement and partnerships
Dimitropoulos 2020 [73]	Implementation	Community Dent Health	NSW	Rural, Remote	Difficulties achieving the target sample size	Community engagement and partnerships, sample qualities, Aboriginal and Torres Strait Islander research team, culturally appropriate and safe research practice, capacity building efforts
Dingwall 2017 [74]	Pilot intervention	BMC Psychol	SA, WA, NT	Remote	Difficulties achieving the target sample size	
Divaris 2013 [75]	Trial	Caries Res	NT	Not stated		
Dorrington 2015 [76]	Evaluation	Aust J Prim Health	ACT	Urban	Inadequate time to conduct research	Community engagement and partnerships
Doyle 2016 [77]	Evaluation	BMC Health Serv Res	VIC	Not stated	Difficulties achieving the target sample size	Community engagement and partnerships, understanding local culture and context
Duley 2017 [78]	Evaluation	Health Promot J Austr	NSW	Regional, Remote, Urban	Difficulties achieving the target sample size	Community engagement and partnerships, culturally appropriate and safe research practice
Durey 2017 [79]	Evaluation	BMC Health Serv Res	WA	Not stated	Difficulties achieving the target sample size	
Eades 2012 [80]	Trial	Med J Aust	QLD	Urban	Difficulties achieving the target sample size	
Edmunds 2016 [81]	Evaluation	BMC Pregnancy Childbirth	QLD	Remote		
Eley 2010a [82]	Evaluation	J Rural Health	QLD	Not stated	Funding/costs/resources	
Eley 2010b [83]	Evaluation	Health Promot J Austr	QLD	Rural		Community engagement and partnerships, sample qualities
Elliott 2010 [84]	Pilot intervention	Telemed J E Health	QLD	Remote		Community engagement and partnerships, Aboriginal and Torres Strait Islander research team, culturally appropriate and safe research practice
Farnbach 2019 [85]	Evaluation	BMC Public Health	National	National		

Ferson 2019 [86]	Pilot intervention	Commun Dis Intell	NSW	Urban		
Fletcher 2015 [87]	Implementation	Int J Lang Commun Disord	NT	Remote	Difficulties achieving the target sample size	Culturally appropriate and safe research practice
Genat 2016 [88]	Evaluation	Contemp Nurse	VIC	Other		Community engagement and partnerships, Aboriginal and Torres Strait Islander research team, capacity building efforts
Gibson-Helm 2018 [89]	Evaluation	PLoS One	National	Rural, Remote, Urban		Sample qualities
Gordon 2012 [90]	Evaluation	N S W Public Health Bull	NSW	Not stated		
Gould 2017 [91]	Implementation	Implement Sci	National	Other		
Gould 2019 [92]	Pilot intervention	Addict Behav	NSW, SA, QLD	Other	Difficulties achieving the target sample size, inadequate time to conduct research	Sample qualities, understanding local culture and context
Govil 2014 [93]	Evaluation	Aust J Prim Health	WA	Regional	Difficulties achieving the target sample size	
Graham 2015 [94]	Evaluation	BMC Infect Dis	NSW	Not stated		Sample qualities
Guy 2018 [95]	Trial	Lancet Infect Dis	SA, WA, QLD	Rural, Remote		Sample qualities
Guy 2015 [96]	Trial	Sex Transm Infect	NT	Remote	Difficulties achieving the target sample size	Sample qualities
Haag 2019 [97]	Trial	Nutrients	SA	Not stated	Difficulties achieving the target sample size	
Haigh 2016 [98]	Evaluation	Aust N Z J Public Health	WA, QLD	Not stated	Difficulties achieving the target sample size	
Harch 2012 [99]	Evaluation	Aust Fam Physician	WA	Remote		Providing resources or reducing costs for services and communities
Hare 2015 [100]	Trial	Eur J Clin Microbiol Infect Dis	NT	Remote	Difficulties achieving the target sample size	
Haynes 2019 [101]	Evaluation	Eval Program Plann	NT	Remote		Community engagement and partnerships
Hearn 2011 [102]	Evaluation	Health Promot J Austr	NSW	Not stated	Difficulties achieving the target sample size	
Hickey 2019 [103]	Evaluation	Eval Program Plann	National	Urban		Community engagement and partnerships

Hogg 2017 [104]	Evaluation	JBI Database System Rev Implement Rep	QLD	Urban	Difficulties achieving the target sample size, inadequate time to conduct research	Community engagement and partnerships, Aboriginal and Torres Strait Islander research team
Hotu 2018 [105]	Evaluation	Aust J Rural Health	National	Remote	Difficulties achieving the target sample size	
Hoy 2019 [106]	Trial	Contemp Clin Trials Commun	NT	Remote	Difficulties achieving the target sample size, inadequate time to conduct research	
Hu 2019a [107]	Implementation	Public Health	QLD	Regional, Urban		
Hu 2019b [108]	Implementation	Aust J Prim Health	QLD	Urban	Difficulties achieving the target sample size	Community engagement and partnerships, culturally appropriate and safe research practice
Hume 2014 [109]	Pilot intervention	Aust N Z J Public Health	NT	Remote	Difficulties achieving the target sample size	Providing resources or reducing costs for services and communities
Isaacs 2014 [110]	Implementation	Australas Psychiatry	VIC	Rural, Remote	Difficulties achieving the target sample size, limited capacity of health workers and services	Community engagement and partnerships
Ivers 2019 [111]	Evaluation	Aust J Rural Health	NSW	Not stated	Difficulties achieving the target sample size	
Jacups 2018 [112]	Evaluation	J Eval Clin Pract	QLD	Remote		
Jainullabudeen 2015 [113]	Implementation	BMC Public Health	QLD	Not stated		Community engagement and partnerships
Jamieson 2016 [114]	Trial	J Health Care Poor Underserved	SA	Not stated		
Jamieson 2020 [115]	Implementation	BMC Res Notes	NT	Remote	Difficulties achieving the target sample size, lack of community involvement	
Jamieson 2019a [116]	Trial	JAMA Netw Open	SA	Other		Sample qualities
Jamieson 2019b [117]	Trial	JAMA Netw Open	SA	Other	Difficulties achieving the target sample size	
Jamieson 2018 [118]	Trial	EClinicalMedicine	SA	Regional, Urban		Community engagement and partnerships
Janca 2015 [119]	Evaluation	Australas Psychiatry	WA, NT	Rural, Remote, Urban	Difficulties achieving the target sample size	
Janca 2017 [120]	Evaluation	Australas Psychiatry	National	National	Difficulties achieving the target sample size	
Johnson 2015 [121]	Trial	BMC Public Health	QLD	Remote	Difficulties achieving the target sample size	

Ju 2017 [122]	Trial	Community Dent Oral Epidemiol	SA	Regional	Difficulties achieving the target sample size	Community engagement and partnerships
Kanagasingam 2015 [123]	Implementation	Conf Proc IEEE Eng Med Biol Soc	WA, QLD	Rural, Remote		
Kapellas 2013 [124]	Trial	J Clin Periodontol	NT	Other	Difficulties achieving the target sample size	Community engagement and partnerships, sample qualities
Kapellas 2014 [125]	Trial	Hypertension	NT	Other	Difficulties achieving the target sample size	
Kapellas 2017 [126]	Trial	Int J Dent Hyg	NT	Regional, Urban		
Khalil 2019 [127]	Implementation	Aust J Rural Health	Other	Rural	Difficulties achieving the target sample size	Community engagement and partnerships, providing resources or reducing costs for services and communities
Kiran 2010 [128]	Trial	Health Promot J Austr	QLD	Not stated		
Kirkham 2019 [129]	Pilot intervention	Aust N Z J Obstet Gynaecol	NT	Remote	Difficulties achieving the target sample size, limited capacity of health workers and services	
Lange 2014 [130]	Implementation	Aust J Prim Health	NT	Remote		
Lange 2017 [131]	Evaluation	PLoS Negl Trop Dis	NT	Remote	Difficulties achieving the target sample size, funding/costs/resources	Sample qualities
Leach 2008a [132]	Trial	BMC Pediatr	NT	Not stated		
Leach 2008b [133]	Trial	Pediatr Infect Dis J	Other	Remote		
Lee 2016 [134]	Pilot intervention	Public Health Res Pract	NSW	Regional, Rural, Urban		
Lee 2008 [135]	Evaluation	Drug Alcohol Rev	NT	Remote		Community engagement and partnerships
Liaw 2015 [136]	Pilot intervention	Aust Fam Physician	NSW	Not stated	Difficulties achieving the target sample size	
Liaw 2019 [137]	Trial	Med J Aust	NSW, VIC	Urban	Difficulties achieving the target sample size, inadequate time to conduct research	
Liberato 2016 [138]	Pilot intervention	Aust N Z J Public Health	NT	Remote	Difficulties achieving the target sample size	
Lin 2016 [139]	Pilot intervention	BMC Fam Pract	WA	Rural	Difficulties achieving the target sample size	

Lobo 2020 [140]	Evaluation	Sex Health		Remote		Community engagement and partnerships, Aboriginal and Torres Strait Islander research team, capacity building efforts
LoGiudice 2012 [141]	Implementation	Rural Remote Health	WA	Remote	Funding/costs/resources	Community engagement and partnerships, Aboriginal and Torres Strait Islander research team, providing resources or reducing costs for services and communities
Lovie-Toon 2018 [142]	Evaluation	Front Pediatr	QLD	Urban	Difficulties achieving the target sample size	Appropriate timelines for completion
Lowell 2015 [143]	Evaluation	BMC Pregnancy Childbirth	NT	Remote	Difficulties achieving the target sample size, funding/costs/resources, communication issues between communities and researchers	Community engagement and partnerships, Aboriginal and Torres Strait Islander research team
Lukaszyk 2018 [144]	Pilot intervention	Health Promot J Austr	NSW	Not stated	Inadequate time to conduct research	Community engagement and partnerships
MacDonald 2016 [145]	Pilot intervention	Aust J Prim Health	VIC	Regional, Rural, Urban	Difficulties achieving the target sample size, limited capacity of health workers and services	Community engagement and partnerships
Macniven 2019 [146]	Pilot intervention	Aust N Z J Public Health	NSW, WA, NT	Rural, Remote, Urban	Inadequate time to conduct research, limited capacity of health workers and services	Community engagement and partnerships, sample qualities
Magnus 2018 [147]	Evaluation	PLoS One	NT	Remote		
Maksimovic 2015 [148]	Evaluation	Health Promot J Austr	SA	Other	Difficulties achieving the target sample size, inadequate time to conduct research, funding/costs/resources	Community engagement and partnerships, sample qualities
Malseed 2014 [149]	Evaluation	Aust J Prim Health	QLD	Urban	Difficulties achieving the target sample size, inadequate time to conduct research	Sample qualities
Manifold 2019 [150]	Pilot intervention	Aust J Prim Health	WA	Remote	Difficulties achieving the target sample size	
Marley 2014 [151]	Trial	BMC Public Health	WA	Remote	Difficulties achieving the target sample size	
Martin 2019 [152]	Pilot intervention	Aust N Z J Public Health	National	Not stated	Difficulties achieving the target sample size, funding/costs/resources, communication issues between communities and researchers	Culturally appropriate and safe research practice
McCallum 2015 [153]	Trial	Front Pediatr	NT	Not stated		Sample qualities
McDonald 2015 [154]	Evaluation	BMC Public Health	NSW, SA, WA, NT	Remote	Inadequate time to conduct research	
McHugh 2019 [155]	Trial	Aust N Z J Obstet Gynaecol	NT	Remote, Urban		Sample qualities

McKay 2015 [156]	Evaluation	Health Promot J Austr	NT	Not stated	Difficulties achieving the target sample size	
McMahon 2017 [157]	Implementation	Nutrients	WA, NT	Rural, Remote	Difficulties achieving the target sample size	
McRae 2008 [158]	Evaluation	Rural Remote Health	NSW	Regional	Limited capacity of health workers and services	Community engagement and partnerships
Medlin 2014 [159]	Pilot intervention	Aust Health Rev	QLD	Rural, Remote	Difficulties achieving the target sample size, funding/costs/resources, limited capacity of health workers and services, communication issues between communities and researchers	Community engagement and partnerships, Aboriginal and Torres Strait Islander research team
Meihubers 2013 [160]	Implementation	N S W Public Health Bull	NSW	Regional		Community engagement and partnerships, capacity building efforts
Mendham 2012 [161]	Pilot intervention	Eur J Appl Physiol		Regional		
Mendham 2014 [162]	Pilot intervention	Am J Hum Biol		Regional		
Mendham 2015 [163]	Trial	J Sci Med Sport	NSW	Regional		Community engagement and partnerships
Meyer 2016 [164]	Evaluation	Health Promot J Austr	WA	Regional, Remote, Urban	Difficulties achieving the target sample size	
Mills 2017 [165]	Evaluation	BMC Health Serv Res	QLD	Not stated	Difficulties achieving the target sample size	
Morris 2010 [166]	Trial	Med J Aust	NT	Rural, Remote	Difficulties achieving the target sample size	
Munro 2017 [167]	Evaluation	Aust J Rural Health	NSW	Remote		Understanding local culture and context
Nagel 2009 [168]	Trial	Aust J Rural Health	NT	Remote	Difficulties achieving the target sample size	
Nguyen 2018 [169]	Pilot intervention	Prev Sci	NT	Remote		Community engagement and partnerships, sample qualities, capacity building efforts
Nguyen 2015 [170]	Evaluation	PLoS One	QLD	Not stated		
Noble 2015 [171]	Trial	BMC Fam Pract	NSW	Regional	Difficulties achieving the target sample size	
O'Donoghue 2014 [172]	Evaluation	Aust J Prim Health	NT	Not stated		Sample qualities
O'Grady 2015 [173]	Pilot intervention	BMC Res Notes	QLD	Not stated	Difficulties achieving the target sample size	Culturally appropriate and safe research practice, capacity building efforts,

						Aboriginal and Torres Strait Islander research team
O'Grady 2012 [174]	Trial	Pediatr Pulmonol	NT	Remote		
O'Halloran 2018 [175]	Evaluation	Clin Exp Ophthalmol	WA	Remote, Urban	Difficulties achieving the target sample size	
Ong 2014 [176]	Evaluation	Heart Lung Circ	National	National		Understanding local culture and context
Panaretto 2013 [177]	Evaluation	BMJ Open	QLD	Not stated		
Passmore 2017 [178]	Evaluation	BMC Public Health	NSW	Other	Difficulties achieving the target sample size	Understanding local culture and context, Aboriginal and Torres Strait Islander research team
Payne 2013 [179]	Evaluation	Contemp Nurse	QLD	Not stated		
Peiris 2019a [180]	Evaluation	JMIR Mhealth Uhealth	NSW	Not stated	Difficulties achieving the target sample size	
Peiris 2019b [181]	Trial	JMIR Mhealth Uhealth	NSW	Not stated	Difficulties achieving the target sample size	
Pettigrew 2015 [182]	Evaluation	Soc Sci Med	WA	Regional, Urban		
Phillips 2014 [183]	Trial	J Paediatr Child Health	NT	Remote	Difficulties achieving the target sample size	Community engagement and partnerships
Poder 2019 [184]	Evaluation	Health Promot J Austr	NSW	Not stated	Difficulties achieving the target sample size	Sample qualities, culturally appropriate and safe research practice
Prowse 2014 [185]	Trial	Int J Ment Health Syst	NT	Remote	Difficulties achieving the target sample size	
Quilty 2019 [186]	Pilot intervention	Int J Environ Res Public Health	NT	Rural, Remote	Inadequate time to conduct research, funding/costs/resources	
Quinn 2017 [187]	Evaluation	Int J Equity Health	NSW	Not stated	Difficulties achieving the target sample size	
Raphiphatthana 2020 [188]	Implementation	BMC Health Serv Res	SA, NT	Remote	Difficulties achieving the target sample size	Community engagement and partnerships
Read 2018 [189]	Evaluation	J Am Heart Assoc	NT	Not stated	Difficulties achieving the target sample size	
Reath 2008 [190]	Implementation	Aust Fam Physician	Other	Regional, Rural, Urban		Community engagement and partnerships, culturally appropriate and safe research practice, capacity building efforts

Reeve 2016 [191]	Evaluation	Aust J Rural Health	WA	Remote		
Reeve 2015 [192]	Evaluation	Med J Aust	WA	Remote		
Reeve 2014 [193]	Evaluation	Aust J Rural Health	WA	Remote		
Reilly 2011 [194]	Pilot intervention	BMC Public Health	VIC	Regional, Urban	Difficulties achieving the target sample size, inadequate time to conduct research	
Ritchie 2010 [195]	Trial	J Pediatr Gastroenterol Nutr	NT	Rural, Remote	Difficulties achieving the target sample size	
Roberts 2017 [196]	Evaluation	J Am Heart Assoc	NT	Other		
Robertson 2013 [197]	Implementation	Int J Environ Res Public Health	NT	Remote		Community engagement and partnerships, Appropriate timelines for completion
Roberts-Thomson 2019 [198]	Pilot intervention	Aust Dent J	Other	Remote	Difficulties achieving the target sample size	
Roberts-Thomson 2010 [199]	Trial	Int Dent J	NT	Remote		Community engagement and partnerships, Aboriginal and Torres Strait Islander research team
Robinson 2020 [200]	Pilot intervention	Front Public Health	NT	Remote	Difficulties achieving the target sample size	
Schmidt 2016 [201]	Evaluation	Aust N Z J Public Health	QLD	Remote	Difficulties achieving the target sample size	Sample qualities, capacity building efforts
Schultz 2012 [202]	Implementation	Rural Remote Health	NT	Remote		
Seear 2020 [203]	Implementation	Eval Program Plann	WA	Remote	Difficulties achieving the target sample size, limited capacity of health workers and services	Community engagement and partnerships, Aboriginal and Torres Strait Islander research team, culturally appropriate and safe research practice
Segal 2016 [204]	Trial	Med J Aust	QLD	Rural, Remote	Difficulties achieving the target sample size	
Shephard 2016 [205]	Evaluation	Aust J Prim Health	National	Other	Difficulties achieving the target sample size, funding/costs/resources	
Shephard 2017 [206]	Evaluation	Clin Biochem	National	Other		
Shield 2018 [207]	Implementation	Trop Med Infect Dis	NT	Remote		Community engagement and partnerships
Sinclair 2016 [208]	Evaluation	Aust N Z J Public Health	WA	Remote		

Slade 2011 [209]	Trial	Community Dent Oral Epidemiol	NT	Remote	Difficulties achieving the target sample size, limited capacity of health workers and services	
Smith 2013 [210]	Implementation	J Telemed Telecare	QLD	Remote		Community engagement and partnerships, Aboriginal and Torres Strait Islander research team
Smith 2012 [211]	Implementation	J Telemed Telecare	QLD	Remote		Aboriginal and Torres Strait Islander research team
Smith 2015 [212]	Evaluation	J Telemed Telecare	QLD	Not stated	Inadequate time to conduct research	Community engagement and partnerships, Aboriginal and Torres Strait Islander research team
Smith 2018 [213]	Implementation	Community Dent Health	NSW	Rural, Remote, Urban	Difficulties achieving the target sample size, inadequate time to conduct research	Providing resources or reducing costs for services and communities, capacity building efforts
Smithers 2017 [214]	Trial	Br J Nutr	SA	Rural, Remote		Community engagement and partnerships, Aboriginal and Torres Strait Islander research team
Snodgrass 2020 [215]	Implementation	Aust J Rural Health	NSW, WA, QLD	Regional, Rural	Difficulties achieving the target sample size	
Soares 2020 [216]	Trial	Int J Paediatr Dent	SA	Rural, Urban	Difficulties achieving the target sample size	
Spaeth 2014 [217]	Implementation	Rural Remote Health	NT	Remote		
Spurling 2013 [218]	Evaluation	BMC Med Inform Decis Mak	QLD	Urban	Difficulties achieving the target sample size	
Spurling 2009 [219]	Evaluation	Med J Aust	QLD	Urban	Difficulties achieving the target sample size	Sample qualities
Standen 2020 [220]	Evaluation	Int J Environ Res Public Health	NSW	Not stated		
Stanley 2019 [221]	Evaluation	Australas Psychiatry	WA	Not stated	Difficulties achieving the target sample size	
Stephen 2013 [222]	Trial	Med J Aust	NT	Remote	Difficulties achieving the target sample size	Sample qualities
Sun 2016 [223]	Trial	Am J Health Promot	QLD	Not stated	Difficulties achieving the target sample size	
Tan 2015 [224]	Trial	J Paediatr Child Health	WA	Rural, Urban	Difficulties achieving the target sample size	
Tane 2018 [225]	Evaluation	Health Promot J Austr	NT	Remote	Difficulties achieving the target sample size	Community engagement and partnerships

Thomas 2020 [226]	Evaluation	Tob Control	SA, WA, NT	Remote		
Thomas 2010 [227]	Evaluation	Aust N Z J Public Health	NT	Remote	Difficulties achieving the target sample size	
Thomas 2008 [228]	Evaluation	N S W Public Health Bull	NSW	Urban		
Thornton 2017a [229]	Trial	Int J Pediatr Otorhinolaryngol	WA	Not stated		
Thornton 2017b [230]	Trial	Clin Vaccine Immunol	WA	Not stated		
Tighe 2017 [231]	Trial	BMJ Open	WA	Remote	Difficulties achieving the target sample size	Community engagement and partnerships, sample qualities
Tong 2010 [232]	Trial	J Paediatr Child Health	NT	Remote		
Treloar 2018 [233]	Evaluation	Harm Reduct J	NSW	Urban	Difficulties achieving the target sample size	
Valery 2010 [234]	Trial	Med J Aust	QLD	Remote	Difficulties achieving the target sample size	Sample qualities
Vallesi 2018 [235]	Evaluation	Int J Environ Res Public Health	WA	Not stated		
Ward 2016 [236]	Trial	Med J Aust	WA, NT, QLD	Remote		Sample qualities
Ward 2019 [237]	Trial	Lancet Glob Health	WA, NT, QLD	Remote		
Whiteside 2012 [238]	Evaluation	Health Promot J Austr	QLD	Remote		
Xu 2018 [239]	Evaluation	Aust J Prim Health	VIC	Not stated	Difficulties achieving the target sample size	
Young 2016 [240]	Evaluation	Aust N Z J Public Health	NSW	Urban	Inadequate time to conduct research	

Note. NSW = New South Wales, QLD = Queensland, VIC = Victoria, NT = Northern Territory, SA = South Australia, WA = Western Australia, ACT = Australian National Territory, TAS = Tasmania.
** The articles included in the original systematic review ranged from 2008-2020. This article was included in the original review as a preprint from 2020, however; it was since fully published in 2021.*

References

1. Abbott, P.; Menzies, R.; Davison, J.; Moore, L.; Wang, H. Improving immunisation timeliness in Aboriginal children through personalised calendars. *BMC Public Health* **2013**, *13*, 598.
2. Adams, K.; Burns, C.; Liebzeit, A.; Ryschka, J.; Thorpe, S.; Browne, J. Use of participatory research and photo-voice to support urban Aboriginal healthy eating. *Health Soc. Care Community* **2012**, *20*, 497–505.
3. Andrews, R.M.; Kearns, T.; Connors, C.; Parker, C.; Carville, K.; Currie, B.J.; Carapetis, J.R. A regional initiative to reduce skin infections amongst aboriginal children living in remote communities of the Northern Territory, Australia. *PLoS Negl. Trop. Dis.* **2009**, *3*, e554.
4. Anikeeva, O.; Katterl, R.; Bywood, P. The Closing the Gap Initiative—Successes and ongoing challenges for divisions of general practice. *Aust. Fam. Physician.* **2012**, *41*, 523–527.
5. Anstey, K.J.; Kiely, K.M.; Booth, H.; Birrell, C.L.; Butterworth, P.; Byles, J.; Luszcz, M.A.; Gibson, R. Indigenous Australians are under-represented in longitudinal ageing studies. *Aust. N. Z. J. Public Health* **2011**, *35*, 331–336.
6. Armstrong, G.; Sutherland, G.; Pross, E.; Mackinnon, A.; Reavley, N.; Jorm, A.F. Talking about suicide: An uncontrolled trial of the effects of an Aboriginal and Torres Strait Islander mental health first aid program on knowledge, attitudes and intended and actual assisting actions. *PLoS One* **2020**, *15*, e0244091.
7. Arrow, P.; Piggott, S.; Carter, S.; McPhee, R.; Atkinson, D.; Mackean, T.; Kularatna, S.; Tonmukayakul, U.; Brennan, D.; Nanda, S.; et al. Atraumatic restorative treatments in Australian Aboriginal communities: A cluster-randomized trial. *JDR Clin. Trans. Res.* **2021**, *6*, 430–439.
8. Askew, D.A.; Guy, J.; Lyall, V.; Egert, S.; Rogers, L.; Pokino, L.A.; Manton-Williams, P.; Schluter, P.J. A mixed methods exploratory study tackling smoking during pregnancy in an urban Aboriginal and Torres Strait Islander primary health care service. *BMC Public Health* **2019**, *19*, 343.
9. Askew, D.A.; Togni, S.J.; Schluter, P.J.; Rogers, L.; Egert, S.; Potter, N.; Hayman, N.E.; Cass, A.; Brown, A.D.H. Investigating the feasibility, acceptability and appropriateness of outreach case management in an urban Aboriginal and Torres Strait Islander primary health care service: A mixed methods exploratory study. *BMC Health Serv. Res.* **2016**, *16*, 178.
10. Atkinson, J.R.; Boudville, A.I.; Stanford, E.E.; Lange, F.D.; Anjou, M.D.. Australian Football League clinics promoting health, hygiene and trachoma elimination: The Northern Territory experience. *Aust. J. Prim. Health* **2014**, *20*, 334–338.
11. Bailie, J.; Schierhout, G.; Laycock, A.; Kelaher, M.; Percival, N.; O'Donoghue, L.; McNeair, T.; Bailie, R. Determinants of access to chronic illness care: A mixed-methods evaluation of a national multifaceted chronic disease package for Indigenous Australians. *BMJ Open* **2015**, *5*, e008103.
12. Bailie, R.S.; McDonald, E.L.; Stevens, M.; Guthridge, S.; Brewster, D.R. Evaluation of an Australian indigenous housing programme: Community level impact on crowding, infrastructure function and hygiene. *J. Epidemiol. Community Health* **2011**, *65*, 432–437.
13. Barrett, E.; Salem, L.; Wilson, S.; O'Neill, C.; Davis, K.; Bagnulo, S. Chronic kidney disease in an Aboriginal population: A nurse practitioner-led approach to management. *Aust. J. Rural. Health* **2015**, *23*, 318–321.
14. Bar-Zeev, Y.; Bovill, M.; Bonevski, B.; Gruppetta, M.; Oldmeadow, C.; Palazzi, K.; Atkins, L.; Reath, J.; Gould, G.S. Improving smoking cessation care in pregnancy at Aboriginal Medical Services: 'ICAN QUIT in Pregnancy' step-wedge cluster randomised study. *BMJ Open* **2019**, *9*, e025293.
15. Battersby, M.; Lawn, S.; Kowanko, I.; Bertossa, S.; Trowbridge, C.; Liddicoat, R. Chronic condition self-management support for Aboriginal people: Adapting tools and training. *Aust. J. Rural. Health* **2018**, *26*, 232–237.
16. Bennett-Levy, J.; Singer, J.; DuBois, S.; Hyde, K. Translating e-mental health into practice: What are the barriers and enablers to e-mental health implementation by Aboriginal and Torres Strait Islander health professionals? *J. Med. Internet. Res.* **2017**, *19*, e1.
17. Berends, L.; Halliday, R. Capacity building and social marketing promotes healthy lifestyle behaviour in an Australian Aboriginal community. *Aust. J. Rural. Health* **2018**, *26*, 279–283.
18. Bernardes, C.M.; Martin, J.; Cole, P.; Kitchener, T.; Cowburn, G.; Garvey, G.; Walpole, E.; Valery, P.C. Lessons learned from a pilot study of an Indigenous patient navigator intervention in Queensland, Australia. *Eur. J. Cancer Care* **2018**, *27*, e12714.
19. Bertilone, C.; McEvoy, S. Success in Closing the Gap: Favourable neonatal outcomes in a metropolitan Aboriginal Maternity Group Practice Program. *Med. J. Aust.* **2015**, *203*, 262.
20. Bierbaum, M.; Plueckhahn, T.; Roth, F.; McNamara, C.; Ramsey, I.; Corsini, N. Challenges to uptake of cancer education resources by rural Aboriginal Health Workers: The Cancer Healing Messages flipchart experience. *Rural. Remote Health* **2017**, *17*, 4199.

21. Biggs, K.; Walsh, J.; Ooi, C. Deadly Liver Mob: Opening the door—Improving sexual health pathways for Aboriginal people in Western Sydney. *Sex Health* **2016**, *13*, 457–464.
22. Binks, M.J.; Moberley, S.A.; Balloch, A.; Leach, A.J.; Nelson, S.; Hare, K.M.; Wilson, C.; Morris, P.S.; Nelson, J.; Chatfield, M.D.; et al. PneuMum: Impact from a randomised controlled trial of maternal 23-valent pneumococcal polysaccharide vaccination on middle ear disease amongst Indigenous infants, Northern Territory, Australia. *Vaccine* **2015**, *33*, 6579–6587.
23. Binks, M.J.; Moberley, S.A.; Balloch, A.; Leach, A.J.; Nelson, S.; Hare, K.M.; Wilson, C.; Nelson, J.; Morris, P.S.; Ware, R.S.; et al. Impact of the 23-valent pneumococcal polysaccharide vaccination in pregnancy against infant acute lower respiratory infections in the Northern Territory of Australia. *Pneumonia* **2018**, *10*, 13.
24. Bird, K.; Fitts, M.S.; Clough, A.R. Alcohol management plans in Indigenous communities in Queensland (Australia) may have unintended implications for the care of children. *Health Justice* **2016**, *4*, 8.
25. Black, A.P.; Vally, H.; Morris, P.; Daniel, M.; Esterman, A.; Karschimkus, C.S.; O'Dea, K. Nutritional impacts of a fruit and vegetable subsidy programme for disadvantaged Australian Aboriginal children. *Br. J. Nutr.* **2013**, *110*, 2309–2317.
26. Black, A.P.; Vally, H.; Morris, P.S.; Daniel, M.; Esterman, A.J.; Smith, F.E.; O'Dea, K. Health outcomes of a subsidised fruit and vegetable program for Aboriginal children in northern New South Wales. *Med. J. Aust.* **2013**, *199*, 46–50.
27. Black, A.P.; Vally, H.; Morris, P.; Daniel, M.; Esterman, A.; Smith, F.; O'Dea, K. High folate levels in Aboriginal children after subsidised fruit and vegetables and mandatory folic acid fortification. *Aust. N. Z. J. Public Health* **2014**, *38*, 241–246.
28. Blignault, I.; Haswell, M.; Jackson Pulver, L. The value of partnerships: Lessons from a multi-site evaluation of a national social and emotional wellbeing program for Indigenous youth. *Aust. N. Z. J. Public Health* **2016**, *40* (Suppl. S1), S53–S58.
29. Borg, K.; Sutton, K.; Beasley, M.; Tull, F.; Faulkner, N.; Halliday, J.; Knott, C.; Bragge, P. Communication-based interventions for increasing influenza vaccination rates among Aboriginal children: A randomised controlled trial. *Vaccine* **2018**, *36*, 6790–6795.
30. Bowen, A.C.; Harris, T.; Holt, D.C.; Giffard, P.M.; Carapetis, J.R.; Campbell, P.T.; McVernon, J.; Tong, S.Y.. Whole genome sequencing reveals extensive community-level transmission of group A Streptococcus in remote communities. *Epidemiol. Infect.* **2016**, *144*, 1991–1998.
31. Bowen, A.C.; Tong, S.Y.; Chatfield, M.D.; Carapetis, J.R. The microbiology of impetigo in indigenous children: Associations between Streptococcus pyogenes, Staphylococcus aureus, scabies, and nasal carriage. *BMC Infect. Dis.* **2014**, *14*, 727.
32. Boyle, J.; Hollands, G.; Beck, S.; Hampel, G.; Wapau, H.; Arnot, M.; Browne, L.; Teede, H.J.; Moran, L.J. Process evaluation of a pilot evidence-based Polycystic Ovary Syndrome clinic in the Torres Strait. *Aust. J. Rural. Health* **2017**, *25*, 175–181.
33. Bradley, C.; Hengel, B.; Crawford, K.; Elliott, S.; Donovan, B.; Mak, D.B.; Nattabi, B.; Johnson, D.; Guy, R.; Fairley, C.K.; et al. Establishment of a sentinel surveillance network for sexually transmissible infections and blood borne viruses in Aboriginal primary care services across Australia: The ATLAS project. *BMC Health Serv. Res.* **2020**, *20*, 769.
34. Bradshaw, S.; Hellwig, L.; Peate, D.; Wilson, A. Promoting the uptake of preventative Aboriginal child health policy in Western Australia. *Aust. J. Rural. Health* 2015, 23, 313–317.
35. Brimblecombe, J.; Bailie, R.; van den Boogaard, C.; Wood, B.; Liberato, S.C.; Ferguson, M.; Coveney, J.; Jaenke, R.; Ritchie, J. Feasibility of a novel participatory multi-sector continuous improvement approach to enhance food security in remote Indigenous Australian communities. *SSM Popul. Health* **2017**, *3*, 566–576.
36. Brimblecombe, J.; Ferguson, M.; Barzi, F.; Brown, C.; Ball, K. Mediators and moderators of nutrition intervention effects in remote Indigenous Australia. *Br. J. Nutr.* **2018**, *119*, 1424–1433.
37. Brimblecombe, J.; Ferguson, M.; Chatfield, M.D.; Liberato, S.C.; Gunther, A.; Ball, K.; Moodie, M.; Miles, E.; Magnus, A.; Mhurchu, C.N.; et al. Effect of a price discount and consumer education strategy on food and beverage purchases in remote Indigenous Australia: A stepped-wedge randomised controlled trial. *Lancet Public Health* **2017**, *2*, e82–e95.
38. Brown, C.; Laws, C.; Leonard, D.; Campbell, S.; Merone, L.; Hammond, M.; Thompson, K.; Canuto, K.; Brimblecombe, J. Healthy Choice Rewards: A feasibility trial of incentives to influence consumer food choices in a remote Australian Aboriginal community. *Int. J. Environ. Res. Public Health* **2019**, *16*, 112.
39. Browne, J.; Gleeson, D.; Adams, K.; Minniecon, D.; Hayes, R. Strengthening Aboriginal and Torres Strait Islander health policy: Lessons from a case study of food and nutrition. *Public Health Nutr.* **2019**, *22*, 2868–2878.
40. Browne, J.; D'Amico, E.; Thorpe, S.; Mitchell, C. Feltman: Evaluating the acceptability of a diabetes education tool for Aboriginal health workers. *Aust. J. Prim. Health* **2014**, *20*, 319–322.

41. Browne, J.; MacDonald, C.; Egan, M.; Delbridge, R.; McAleese, A.; Morley, B.; Atkinson, P. You wouldn't eat 16 teaspoons of sugar-so why drink it? Aboriginal and Torres Strait Islander responses to the LiveLighter sugary drink campaign. *Health Promot. J. Austr.* **2019**, *30*, 212–218.
42. Burgess P, Mileran A, Bailie, R. Beyond the mainstream - health gains in remote aboriginal communities. Aust Fam Physician 2008; 37: 986-988.
43. Cameron, S.; Dillon, H.; Glyde, H.; Kanthan, S.; Kania, A. Prevalence and remediation of spatial processing disorder (SPD) in Indigenous children in regional Australia. *Int. J. Audiol.* **2014**, *53*, 326–335.
44. Campbell, M.A.; Finlay, S.; Lucas, K.; Neal, N.; Williams, R. Kick the habit: A social marketing campaign by Aboriginal communities in NSW. *Aust. J. Prim. Health* **2014**, *20*, 327–333.
45. Campbell, S.; McCalman, J.; Redman-MacLaren, M.; Canuto, K.; Vine, K.; Sewter, J.; McDonald, M. Implementing the Baby One Program: A qualitative evaluation of family-centred child health promotion in remote Australian Aboriginal communities. *BMC Pregnancy Childbirth* **2018**, *18*, 73.
46. Canuto, K.J.; Spagnoletti, B.; McDermott, R.A.; Cargo, M. Factors influencing attendance in a structured physical activity program for Aboriginal and Torres Strait Islander women in an urban setting: A mixed methods process evaluation. *Int. J. Equity Health* **2013**, *12*, 11.
47. Canuto, K.; Cargo, M.; Li, M.; D'Onise, K.; Esterman, A.; McDermott, R. Pragmatic randomised trial of a 12-week exercise and nutrition program for Aboriginal and Torres Strait Islander women: Clinical results immediate post and 3 months follow-up. *BMC Public Health* **2012**, *12*, 993.
48. Cargo, M.; Marks, E.; Brimblecombe, J.; Scarlett, M.; Maypilama, E.; Dhurrkay, J.G.; Daniel, M. Integrating an ecological approach into an Aboriginal community-based chronic disease prevention program: A longitudinal process evaluation. *BMC Public Health* **2011**, *11*, 299.
49. Cartwright, K.; Gray, D.; Fewings, E. Demonstrating Impact: Lessons learned from the Queensland Aboriginal and Islander Health Council;s AOD-Our-Way program. *Int. J. Environ. Res. Public Health* **2018**, *15*, 450.
50. Cashman, P.M.; Allan, N.A.; Clark, K.K.; Butler, M.T.; Massey, P.D.; Durrheim, D.N. Closing the gap in Australian Aboriginal infant immunisation rates—The development and review of a pre-call strategy. *BMC Public Health* **2016**, *16*, 514.
51. Causer, L.M.; Hengel, B.; Natoli, L.; Tangey, A.; Badman, S.G.; Tabrizi, S.N.; Whiley, D.; Ward, J.; Kaldor, J.M.; Guy, R.J. A field evaluation of a new molecular-based point-of-care test for chlamydia and gonorrhoea in remote Aboriginal health services in Australia. *Sex Health* **2015**, *12*, 27–33.
52. Chambers, A.H.; Tomnay, J.; Stephens, K.; Crouch, A.; Whiteside, M.; Love, P.; McIntosh, L.; Waples Crowe, P. Facilitators of community participation in an Aboriginal sexual health promotion initiative. *Rural Remote Health* **2018**, *18*, 4245.
53. Chenhall, R. What's in a rehab? Ethnographic evaluation research in Indigenous Australian residential alcohol and drug rehabilitation centres. *Anthropol. Med.* **2008**, *15*, 105–116.
54. Christou, A.; Thompson, S.C. Missed opportunities in educating Aboriginal Australians about bowel cancer screening: Whose job is it anyway? *Contemp. Nurse* **2013**, *46*, 59–69.
55. Ciccone, N.; Armstrong, E.; Hersh, D.; Adams, M.; MCAllister, M. The Wangi (talking) project: A feasibility study of a rehabilitation model for aboriginal people with acquired communication disorders after stroke. *Int. J. Speech Lang Pathol.* **2019**, *21*, 305–316.
56. Clapham, K.; Bennett-Brook, K.; Hunter, K. The role of Aboriginal family workers in delivering a child safety-focused home visiting program for Aboriginal families in an urban region of New South Wales. *Health Promot. J. Austr.* **2018**, *29*, 173–182.
57. Clark, R.A.; Fredericks, B.; Buitendyk, N.J.; Adams, M.J.; Howie-Esquivel, J.; Dracup, K.A.; Berry, N.M.; Atherton, J.; Johnson, S. Development and feasibility testing of an education program to improve knowledge and self-care among Aboriginal and Torres Strait Islander patients with heart failure. *Rural. Remote Health* **2015**, *15*, 38–50.
58. Clifford, A.; Shakeshaft, A. Evidence-based alcohol screening and brief intervention in Aboriginal Community Controlled Health Services: Experiences of health-care providers. *Drug Alcohol Rev.* **2011**, *30*, 55–62.
59. Clifford, A.; Shakeshaft, A.; Deans, C. Training and tailored outreach support to improve alcohol screening and brief intervention in Aboriginal Community Controlled Health Services. *Drug Alcohol Rev.* **2013**, *32*, 72–79.
60. Coffin, J. The Nguudu Barndimanmanha project—Improving social and emotional wellbeing in Aboriginal youth through equine assisted learning. *Front. Public Health* **2019**, *7*, 278.
61. Conigrave, K.; Freeman, B.; Caroll, T.; Simpson, L.; Lee, K.; Wade, V.; Kiel, K.; Ella, S.; Becker, K.; Freeburn, B. The Alcohol Awareness project: Community education and brief intervention in an urban Aboriginal setting. *Health Promot. J. Austr.* **2012**, *23*, 219–225.

62. Cooper, P.; Kohler, M.; Blunden, S. Sleep and academic performance in Indigenous Australian children from a remote community: An exploratory study. *J. Paediatr. Child Health* **2012**, *48*, 122–127.
63. Cresp, R.; Clarke, K.; McAuley, K.E.; McAullay, D.; Moylan, C.A.; Peter, S.; Chaney, G.M.; Cook, A.; Edmond, K.M. Effectiveness of the Koorliny Moort out-of-hospital health care program for Aboriginal and Torres Strait Islander children in Western Australia. *Med. J. Aust.* **2016**, *204*, 234–237.
64. Crouch, A.; Robertson, H.; Fagan, P. Hip hopping the gap—Performing arts approaches to sexual health disadvantage in young people in remote settings. *Australas. Psychiatr.* **2011**, *19* (Suppl S1), S34–S37.
65. Cuesta-Briand, B.; Bessarab, D.; Shahid, S.; Thompson, S.C. Addressing unresolved tensions to build effective partnerships: Lessons from an Aboriginal cancer support network. *Int. J. Equity Health* **2015**, *14*, 123.
66. D'Abbs, P.; Schmidt, B.; Dougherty, K.; Senior, K. Implementing a chronic disease strategy in two remote Indigenous Australian settings: A multi-method pilot evaluation. *Aust. J. Rural. Health* **2008**, *16*, 67–74.
67. Davey, M.; Moore, W.; Walters, J. Tasmanian Aborigines step up to health: Evaluation of a cardiopulmonary rehabilitation and secondary prevention program. *BMC Health Serv. Res.* **2014**, *14*, 349.
68. Davies, K.; Read, D.M.Y.; Booth, A.; Turner, N.; Gottschall, K.; Perkins, D. Connecting with social and emotional well-being in rural Australia: An evaluation of 'We-Yarn', an Aboriginal gatekeeper suicide prevention workshop. *Aust. J. Rural. Health* **2020**, *28*, 579–587.
69. Daws, K.; Punch, A.; Winters, M.; Posenelli, S.; Willis, J.; MacIsaac, A.; Rahman, M.A.; Worrall-Carter, L. Implementing a working together model for Aboriginal patients with acute coronary syndrome: An Aboriginal Hospital Liaison Officer and a specialist cardiac nurse working together to improve hospital care. *Aust. Health Rev.* **2014**, *38*, 552–556.
70. Digiacomo, M.; Abbott, P.; Davison, J.; Moore, L.; Davidson, P.M. Facilitating uptake of Aboriginal Adult Health Checks through community engagement and health promotion. *Qual. Prim. Care* **2010**, *18*, 57–64.
71. Dimer, L.; Dowling, T.; Jones, J.; Cheetham, C.; Thomas, T.; Smith, J.; McManus, A.; Maiorana, A.J. Build it and they will come: Outcomes from a successful cardiac rehabilitation program at an Aboriginal Medical Service. *Aust. Health Rev.* **2013**, *37*, 79–82.
72. Dimitropoulos, Y.; Gwynne, K.; Blinkhorn, A.; Holden, A. A school fluoride varnish program for Aboriginal children in rural New South Wales, Australia. *Health Promot. J. Austr.* **2019**, *31*, 172–176.
73. Dimitropoulos, Y.; Holden, A.; Gwynne, K.; Do, L.; Byun, R.; Sohn, W. Outcomes of a co-designed, community-led oral health promotion program for Aboriginal children in rural and remote communities in New South Wales, Australia. *Community Dent. Health* **2020**, *29*, 132–137.
74. Dingwall, K.M.; Gray, A.O.; McCarthy, A.R.; Delima, J.F.; Bowden, S.C. Exploring the reliability and acceptability of cognitive tests for Indigenous Australians: A pilot study. *BMC Psychol.* **2017**, *5*, 1–16.
75. Divaris, K.; Preisser, J.S.; Slade, G.D. Surface-specific efficacy of fluoride varnish in caries prevention in the primary dentition: Results of a community randomized clinical trial. *Caries Res.* **2013**, *47*, 78–87.
76. Dorrington, M.S.; Herceg, A.; Douglas, K.; Tongs, J.; Bookallil, M. Increasing Pap smear rates at an urban Aboriginal Community Controlled Health Service through translational research and continuous quality improvement. *Aust. J. Prim. Health* **2015**, *21*, 417–422.
77. Doyle, J.; Atkinson-Briggs, S.; Atkinson, P.; Firebrace, B.; Calleja, J.; Reilly, R.; Cargo, M.; Riley, T.; Crumpen, T.; Rowley, K. A prospective evaluation of first people's health promotion program design in the 19oulburn-murray rivers region. *BMC Health Serv. Res.* **2016**, *16*, 26.
78. Duley, P.; Botfield, J.R.; Ritter, T.; Wicks, J.; Brassil, A. The Strong Family Program: An innovative model to engage Aboriginal and Torres Strait Islander youth and Elders with reproductive and sexual health community education. *Health Promot. J. Austr.* **2017**, *28*, 132–138.
79. Durey, A.; Halkett, G.; Berg, M.; Lester, L.; Kickett, M. Does one workshop on respecting cultural differences increase health professionals' confidence to improve the care of Australian Aboriginal patients with cancer? An evaluation. *BMC Health Serv. Res.* **2017**, *17*, 660.
80. Eades, S.J.; Sanson-Fisher, R.W.; Wenitong, M.; Panaretto, K.; D'Este, C.; Gilligan, C.; Stewart, J. An intensive smoking intervention for pregnant Aboriginal and Torres Strait Islander women: A randomised controlled trial. *Med. J. Aust.* **2012**, *197*, 42–46.
81. Edmunds, K.; Searles, A.; Neville, J.; Ling, R.; McCalman, J.; Mein, J. Apunipima baby basket program: A retrospective cost study. *BMC Pregnancy Childbirth* **2016**, *16*, 337.
82. Eley, R.; Gorman, D. Didgeridoo playing and singing to support asthma management in Aboriginal Australians. *J. Rural. Health* **2010**, *26*, 100–104.
83. Eley, R.; Gorman, D.; Gately, J. Didgeridoos, songs and boomerangs for asthma management. *Health Promot. J. Austr.* **2010**, *21*, 39–44.
84. Elliott, G.; Smith, A.C.; Bensink, M.E.; Brown, C.; Stewart, C.; Perry, C.; Scuffham, P. The feasibility of a community-based mobile telehealth screening service for Aboriginal and Torres Strait Islander children in Australia. *Telemed. J. E. Health* **2010**, *16*, 950–956.

85. Farnbach, S.; Gee, G.; Eades, A.M.; Evans, J.R.; Fernando, J.; Hammond, B.; Simms, M.; DeMasi, K.; Glozier, N.; Brown, A.; et al. Process evaluation of the Getting it Right study and acceptability and feasibility of screening for depression with the aPHQ-9. *BMC Public Health* **2019**, *19*, 1207.
86. Ferson, M.J.; Ressler, K.A.; Nurkic, A.; Spokes, P.J. Gonorrhoea enhanced surveillance for indigenous status and risk factors in the south-eastern Sydney population. *Commun. Dis. Intell.* **2019**, *43*, 23.
87. Fletcher, J.; Hogben, J.; Neilson, R.; Lalara, R.D.; Reid, C. Examining the quality of phonological representations in Anindilyakwa children in Australia. *Int. J. Lang Commun. Disord.* **2015**, *50*, 842–848.
88. Genat, B.; Browne, J.; Thorpe, S.; MacDonald, C. Sectoral system capacity development in health promotion: Evaluation of an Aboriginal nutrition program. *Contemp. Nurse* **2016**, *27*, 236–242.
89. Gibson-Helm, M.E.; Bailie, J.; Matthews, V.; Laycock, A.F.; Boyle, J.A.; Bailie, R.S. Identifying evidence-practice gaps and strategies for improvement in Aboriginal and Torres Strait Islander maternal health care. *PLoS One* **2018**, *13*, e0192262.
90. Gordon, R.; Richards, N. The Chronic Care for Aboriginal People program in NSW. *NSW Public Health Bull.* **2012**, *23*, 77–80.
91. Gould, G.S.; Bar-Zeev, Y.; Bovill, M.; Atkins, L.; Gruppetta, M.; Clarke, M.J.; Bonevski, B. Designing an implementation intervention with the Behaviour Change Wheel for health provider smoking cessation care for Australian Indigenous pregnant women. *Implement. Sci.* **2017**, *12*, 114.
92. Gould, G.S.; Bovill, M.; Pollock, L.; Bonevski, B.; Gruppetta, M.; Atkins, L.; Carson-Chahhoud, K.; Boydell, K.M.; Gribbin, G.R.; Oldmeadow, C.; et al. Feasibility and acceptability of Indigenous Counselling and Nicotine (ICAN) QUIT in Pregnancy multicomponent implementation intervention and study design for Australian Indigenous pregnant women: A pilot cluster randomised step-wedge trial. *Addict. Behav.* **2019**, *90*, 176–190.
93. Govil, D.; Lin, I.; Dodd, T.; Cox, R.; Moss, P.; Thompson, S.; Maiorana, A. Identifying culturally appropriate strategies for coronary heart disease secondary prevention in a regional Aboriginal Medical Service. *Aust. J. Prim. Health* **2014**, *20*, 266–272.
94. Graham, S.; Guy, R.J.; Wand, H.C.; Kaldor, J.M.; Donovan, B.; Knox, J.; McCowen, D.; Bullen, P.; Booker, J.; O'Brien, C.; A sexual health quality improvement program (SHIMMER) triples chlamydia and gonorrhoea testing rates among young people attending Aboriginal primary health care services in Australia. *BMC Infect. Dis.* **2015**, *15*, 370.
95. Guy, R.J.; Ward, J.; Causer, L.M.; Natoli, L.; Badman, S.G.; Tangey, A.; Hengel, B.; Wand, H.; Whiley, D.; Tabrizi, S.N.; et al. Molecular point-of-care testing for chlamydia and gonorrhoea in Indigenous Australians attending remote primary health services (TTANGO): A cluster-randomised, controlled, crossover trial. *Lancet Infect. Dis.* **2018**, *18*, 1117–1126.
96. Guy, R.; Ward, J.; Wand, H.; Rumbold, A.; Garton, L.; Hengel, B.; Silver, B.; Taylor-Thomson, D.; Knox, J.; McGregor, S.; et al. Coinfection with Chlamydia trachomatis, Neisseria gonorrhoeae and Trichomonas vaginalis: A cross-sectional analysis of positivity and risk factors in remote Australian Aboriginal communities. *Sex Transm. Infect.* **2015**, *91*, 201–206.
97. Haag, D.G.; Jamieson, L.M.; Hedges, J.; Smithers, L.G. Is There an Association between Breastfeeding and Dental Caries among Three-Year-Old Australian Aboriginal Children? *Nutrients* **2019**, *11*, 2811.
98. Haigh, M.; Shahid, S.; O'Connor, K.; Thompson, S.C. Talking about the not talked about: Use of, and reactions to, a DVD promoting bowel cancer screening to Aboriginal people. *Aust. N. Z. J. Public Health* **2016**, *40*, 548–552.
99. Harch, S.; Reeve, D.; Reeve, C. Management of type 2 diabetes—A community partnership approach. *Aust. Fam. Physician.* **2012**, *41*, 73–76.
100. Hare, K.M.; Grimwood, K.; Chang, A.B.; Chatfield, M.D.; Valery, P.C.; Leach, A.J.; Smith-Vaughan, H.C.; Morris, P.S.; Byrnes, C.A.; Torzillo, P.J.; et al. Nasopharyngeal carriage and macrolide resistance in Indigenous children with bronchiectasis randomized to long-term azithromycin or placebo. *Eur. J. Clin. Microbiol. Infect. Dis.* **2015**, *34*, 2275–2285.
101. Haynes, M.; O'Rourke, T.; Nash, D.; Mitchell, A.G.; Phillips, J.; Bessarab, D.; Walker, R.; Cook, J.; Ralph, A.P. Aboriginal and Torres Strait Islander preferences for healthcare settings: Effective use of design images in survey research. *Aust. Health Rev.* **2019**, *44*, 222–227.
102. Hearn, S.; Nancarrow, H.; Rose, M.; Massi, L.; Wise, M.; Conigrave, K.; Barnes, I.; Bauman, A. Evaluating NSW SmokeCheck: A culturally specific smoking cessation training program for health professionals working in Aboriginal health. *Health Promot. J. Austr.* **2011**, *22*, 189–195.
103. Hickey, S.; Couchman, K.; Stapleton, H.; Roe, Y.; Kildea, S. Experiences of health service providers establishing an Aboriginal-Mainstream partnership to improve maternity care for Aboriginal and Torres Strait Islander families in an urban setting. *Eval. Program. Plann.* **2019**, *77*, 101705.

104. Hogg, S.; Roe, Y.; Mills, R. Implementing evidence-based continuous quality improvement strategies in an urban Aboriginal Community Controlled Health Service in South East Queensland: A best practice implementation pilot. *JBI Database System Rev. Implement Rep.* **2017**, *15*, 178–187.
105. Hotu, C.; Remond, M.; Maguire, G.; Ekinci, E.; Cohen, N. Impact of an integrated diabetes service involving specialist outreach and primary health care on risk factors for micro- and macrovascular diabetes complications in remote Indigenous communities in Australia. *Aust. J. Rural. Health* **2018**, *26*, 394–399.
106. Hoy, W.E.; Reid, C.M.; Huq, M.; McLeod, B.J.; Mott, S.A. A randomised controlled trial of potential for pharmacologic prevention of new-onset albuminuria, hypertension and diabetes in a remote Aboriginal Australian community, 2008–2013. *Contemp. Clin. Trials Commun.* **2019**, *14*, 100323.
107. Hu, J.; Basit, T.; Nelson, A.; Bartlett, A. Changes in exercise capacity and anthropometric measures after Work It Out-a holistic chronic disease self-management program for urban Aboriginal and Torres Strait Islander people. *Public Health* **2019**, *174*, 49–55.
108. Hu, J.; Basit, T.; Nelson, A.; Crawford, E.; Turner, L. Does attending Work It Out—A chronic disease self-management program—Affect the use of other health services by urban Aboriginal and Torres Strait Islander people with or at risk of chronic disease? A comparison between program participants and non-participants. *Aust. J. Prim. Health* **2019**, *25*, 464–470.
109. Hume, A.; Wetten, A.; Feeney, C.; Taylor, S.; O'Dea, K.; Brimblecombe, J. Remote school gardens: Exploring a cost-effective and novel way to engage Australian Indigenous students in nutrition and health. *Aust. N. Z. J. Public Health* **2014**, *38*, 235–240.
110. Isaacs, A.; Lampitt, B. The Koorie Men's Health Day: An innovative model for early detection of mental illness among rural Aboriginal men. *Australas Psychiatr.* **2014**, *22*, 56–61.
111. Ivers, R.; Jackson, B.; Levett, T.; Wallace, K.; Winch, S. Home to health care to hospital: Evaluation of a cancer care team based in Australian Aboriginal primary care. *Aust. J. Rural. Health* **2019**, *27*, 88–92.
112. Jacups, S.P.; Kinchin, I.; McConnon, K.M. Ear, nose, and throat surgical access for remote living Indigenous children: What is the least costly model? *J. Eval. Clin. Pract.* **2018**, *24*, 1330–1338.
113. Jainullabudeen, T.A.; Lively, A.; Singleton, M.; Shakeshaft, A.; Tsey, K.; McCalman, J.; Doran, C.; Jacups, S. The impact of a community-based risky drinking intervention (Beat da Binge) on Indigenous young people. *BMC Public Health* **2015**, *15*, 1319.
114. Jamieson, L.; Bradshaw, J.; Lawrence, H.; Broughton, J.; Venner, K. Fidelity of Motivational Interviewing in an early childhood caries intervention involving Indigenous Australian mothers. *J. Health Care Poor Underserved* **2016**, *27*, 125–138.
115. Jamieson, L.M.; Sajiv, C.; Cass, A.; Maple-Brown, L.J.; Skilton, M.R.; Kapellas, K.; Pawar, B.; Arrow, P.; Askie, L.M.; Hoy, W.; et al. *Lessons learned from a periodontal intervention to reduce progressi*on of chronic kidney disease among Aboriginal Australians. *BMC Res. Notes* **2020**, *13*, 1–5.
116. Jamieson, L.; Smithers, L.; Hedges, J.; Mills, H.; Kapellas, K.; Ha, D.; Do, L.; Ju, X. Follow-up of Intervention to Prevent Dental Caries Among Indigenous Children in Australia: A Secondary Analysis of a Randomized Clinical Trial. *JAMA Netw Open* **2019**, *2*, e1915611.
117. Jamieson, L.M.; Smithers, L.G.; Hedges, J.; Aldis, J.; Mills, H.; Kapellas, K.; Lawrence, H.P.; Broughton, J.R.; Ju, X. Follow-up of an Intervention to Reduce Dental Caries in Indigenous Australian Children: A Secondary Analysis of a Randomized Clinical Trial. *JAMA Netw Open* **2019**, *2*, e190648.
118. Jamieson, L.M.; Smithers, L.G.; Hedges, J.; Parker, E.; Mills, H.; Kapellas, K.; Lawrence, H.P.; Broughton, J.R.; Ju, X. Dental Disease Outcomes Following a 2-Year Oral Health Promotion Program for Australian Aboriginal Children and Their Families: A 2-Arm Parallel, Single-blind, Randomised Controlled Trial. *EClinicalMedicine* **2018**, *1*, 43–50.
119. Janca, A.; Lyons, Z.; Balaratnasingam, S.; Parfitt, D.; Davison, S.; Laugharne, J. Here and Now Aboriginal Assessment: Background, development and preliminary evaluation of a culturally appropriate screening tool. *Australas Psychiatr.* **2015**, *23*, 287–292.
120. Janca, A.; Lyons, Z.; Gaspar, J. Here and Now Aboriginal Assessment (HANAA): A follow-up survey of users. *Australas Psychiatr.* **2017**, *25*, 288–289.
121. Johnson, D.R.; McDermott, R.A.; Clifton, P.M.; D'Onise, K.; Taylor, S.M.; Preece, C.L.; Schmidt, B.A. Characteristics of Indigenous adults with poorly controlled diabetes in north Queensland: Implications for services. *BMC Public Health* **2015**, *15*, 325.
122. Ju, X.; Brennan, D.; Parker, E. Efficacy of an oral health literacy intervention among Indigenous Australian adults. Community *Dent. Oral. Epidemiol.* **2017**, *45*, 413–426.
123. Kanagasingam, Y.; Boyle, J.; Vignarajan, J.; Di, Xiao; Ming, Zhang Establishing an indigenous tele-eye care service. *Conf. Proc. IEEE Eng. Med. Biol. Soc.* **2015**, *2015*, 1608–1611.

124. Kapellas, K.; Do, L.G.; Bartold, P.M.; Skilton, M.R.; Maple-Brown, L.J.; O'Dea, K.; Brown, A.; Celermajer, D.S.; Slade, G.D.; Jamieson, L.M. Effects of full-mouth scaling on the periodontal health of Indigenous Australians: A randomized controlled trial. *J. Clin. Periodontol.* **2013**, *40*, 1016–1024.
125. Kapellas, K.; Maple-Brown, L.J.; Jamieson, L.M.; Do, L.G.; O'Dea, K.; Brown, A.; Cai, T.Y.; Anstey, N.M.; Sullivan, D.R.; Wang, H.; Celermajer, D.S.; et al. Effect of periodontal therapy on arterial structure and function among aboriginal australians: A randomized, controlled trial. *Hypertension* **2014**, *64*, 702–708.
126. Kapellas, K.; Mejia, G.; Bartold, P.M.; Skilton, M.R.; Maple-Brown, L.J.; Slade, G.D.; O'Dea, K.; Brown, A.; Celermajer, D.S.; Jamieson, L.M.; et al. Periodontal therapy and glycaemic control among individuals with type 2 diabetes: Reflections from the PerioCardio study. *Int. J. Dent. Hyg.* **2017**, *15*, e42–e51.
127. Khalil, H. Successful implementation of a medication safety program for Aboriginal Health Practitioners in rural Australia. *Aust. J. Rural. Health* **2019**, *27*, 158–163.
128. Kiran, A.; Knights, J. Traditional Indigenous Games promoting physical activity and cultural connectedness in primary schools—Cluster randomised control trial. *Health Promot. J. Austr.* **2010**, *21*, 149–151.
129. Kirkham, R.; MacKay, D.; Barzi, F.; Whitbread, C.; Kirkwood, M.; Graham, S.; Van Dokkum, P.; McIntyre, H.D.; Shaw, J.E.; Brown, A.; et al. Improving postpartum screening after diabetes in pregnancy: Results of a pilot study in remote Australia. *Aust. N. Z. J. Obstet. Gynaecol.* **2019**, *59*, 430–435.
130. Lange, F.D.; Baunach, E.; McKenzie, R.; Taylor, H.R. Trachoma elimination in remote Indigenous Northern Territory communities: Baseline health-promotion study. *Aust. J. Prim. Health* **2014**, *20*, 34–40.
131. Lange, F.D.; Jones, K.; Ritte, R.; Brown, H.E.; Taylor, H.R. The impact of health promotion on trachoma knowledge, attitudes and practice (KAP) of staff in three work settings in remote Indigenous communities in the Northern Territory. *PLoS Negl. Trop. Dis.* **2017**, *11*, e0005503.
132. Leach, A.; Wood, Y.; Gadil, E.; Stubbs, E.; Morris, P. Topical ciprofloxin versus topical framycetin-gramicidin-dexamethasone in Australian aboriginal children with recently treated chronic suppurative otitis media: A randomized controlled trial. *Pediatr. Infect. Dis. J.* **2008**, *27*, 692–698.
133. Leach, A.J.; Morris, P.S.; Mathews, J.D. Compared to placebo, long-term antibiotics resolve otitis media with effusion (OME) and prevent acute otitis media with perforation (AOMwiP) in a high-risk population: A randomized controlled trial. *BMC Pediatr.* **2008**, *8*, 23.
134. Lee, A.; Lucas, K.; Campbell, M.A.; Sarin, J. Continuing to lift the burden: Using a continuous quality improvement approach to advance Aboriginal tobacco resistance and control. *Public Health Res. Pract.* **2016**, *26*, e2651662.
135. Lee, K.S.; Conigrave, K.M.; Clough, A.R.; Wallace, C.; Silins, E.; Rawles, J. Evaluation of a community-driven preventive youth initiative in Arnhem Land, Northern Territory, Australia. *Drug Alcohol Rev.* **2008**, *27*, 75–82.
136. Liaw, S.T.; Hasan, I.; Wade, V.; Canalese, R.; Kelaher, M.; Lau, P.; Harris, M. Improving cultural respect to improve Aboriginal health in general practice: A multi-methods and multi-perspective pragmatic study. *Aust. Fam. Physician.* **2015**, *44*, 387–392.
137. Liaw, S.T.; Wade, V.; Furler, J.S.; Hasan, I.; Lau, P.; Kelaher, M.; Xuan, W.; Harris, M.F. Cultural respect in general practice: A cluster randomised controlled trial. *Med. J. Aust.* **2019**, *210*, 263–268.
138. Liberato, S.C.; Kearns, T.; Ward, F.; Brimblecombe, J. Use of electronic visual recording to aid assessment of dietary intake of Australian Aboriginal children living in remote communities. *Aust. N. Z. J. Public Health* **2016**, *40* (Suppl S1), S27–S29.
139. Lin, I.B.; Coffin, J.; O'Sullivan, P.B. Using theory to improve low back pain care in Australian Aboriginal primary care: A mixed method single cohort pilot study. *BMC Fam. Pract.* **2016**, *17*, 1–14.
140. Lobo, R.; D'Costa, B.; Forbes, L.; Ward, J. Young Deadly Free: Impact evaluation of a sexual health youth peer education program in remote Australian communities. *Sex Health* **2020**, *17*, 397–404.
141. LoGiudice, D.C.; Smith, K.; Shadforth, G.; Lindeman, M.; Carroll, E.; Atkinson, D.; Schaper, F.; Lautenschlager, N.; Murphy, R.; Flicker, L. Lungurra Ngoora—A pilot model of care for aged and disabled in a remote Aboriginal community—Can it work? *Rural. Remote Health* **2012**, *12*, 2078.
142. Lovie-Toon, Y.G.; McPhail, S.M.; Au-Yeung, Y.T.; Hall, K.K.; Chang, A.B.; Vagenas, D.; Otim, M.E.; O'Grady, K.F. The Cost of Acute Respiratory Infections With Cough Among Urban Aboriginal and Torres Strait Islander Children. *Front. Pediatr.* **2018**, *6*, 379.
143. Lowell, A.; Kildea, S.; Liddle, M.; Cox, B.; Paterson, B. Supporting aboriginal knowledge and practice in health care: Lessons from a qualitative evaluation of the strong women, strong babies, strong culture program. *BMC Pregnancy Childbirth* **2015**, *15*, 19.
144. Lukaszyk, C.; Coombes, J.; Sherrington, C.; Tiedemann, A.; Keay, L.; Mackean, T.; Clemson, L.; Cumming, R.; Broe, T.; Ivers, R. The Ironbark program: Implementation and impact of a community-based fall prevention pilot program for older Aboriginal and Torres Strait Islander people. *Health Promot. J. Austr.* **2018**, *29*, 189–198.

145. MacDonald, C.; Genat, B.; Thorpe, S.; Browne, J. Establishing health-promoting workplaces in Aboriginal community organisations: Healthy eating policies. *Aust. J. Prim. Health* **2016**, *22*, 239–243.
146. Macniven, R.; Gwynn, J.; Fujimoto, H.; Hamilton, S.; Thompson, S.C.; Taylor, K.; Lawrence, M.; Finlayson, H.; Bolton, G.; Dulvari, N.; et al. Feasibility and acceptability of opportunistic screening to detect atrial fibrillation in Aboriginal adults. *Aust. N. Z. J. Public Health* **2019**, *43*, 313–318.
147. Magnus, A.; Cobiac, L.; Brimblecombe, J.; Chatfield, M.; Gunther, A.; Ferguson, M.; Moodie, M. The cost-effectiveness of a 20% price discount on fruit, vegetables, diet drinks and water, trialled in remote Australia to improve Indigenous health. *PLoS One* **2018**, *13*, e0204005.
148. Maksimovic, L.; Shen, D.; Bandick, M.; Ettridge, K.; Eckert, M. Evaluation of the pilot phase of the ;Give up smokes for good; social marketing campaign. *Health Promot. J. Austr.* **2015**, *26*, 16–23.
149. Malseed, C.; Nelson, A.; Ware, R.; Lacey, I.; Lander, K. Deadly Choices community health events: A health promotion initiative for urban Aboriginal and Torres Strait Islander people. *Aust. J. Prim. Health* **2014**, *20*, 379–383.
150. Manifold, A.; Atkinson, D.; Marley, J.V.; Scott, L.; Cleland, G.; Edgill, P.; Singleton, S. Complex diabetes screening guidelines for high-risk adolescent Aboriginal Australians: A barrier to implementation in primary health care. *Aust. J. Prim. Health* **2019**, *25*, 501–508.
151. Marley, J.V.; Atkinson, D.; Kitaura, T.; Nelson, C.; Gray, D.; Metcalf, S.; Maguire, G.P. The Be Our Ally Beat Smoking (BOABS) study, a randomised controlled trial of an intensive smoking cessation intervention in a remote aboriginal Australian health care setting. *BMC Public Health* **2014**, *14*, 32.
152. Martin, K.; Dono, J.; Stewart, H.B.; Sparrow, A.; Miller, C.; Roder, D.; Bowden, J. Evaluation of an intervention to train health professionals working with Aboriginal and Torres Strait Islander people to provide smoking cessation advice. *Aust. N. Z. J. Public Health* **2019**, *43*, 156–162.
153. McCallum, G.B.; Morris, P.S.; Grimwood, K.; Maclennan, C.; White, A.V.; Chatfield, M.D.; Sloots, T.P.; Mackay, I.M.; Smith-Vaughan, H.; McKay, C.C.; et al. Three-weekly doses of azithromycin for indigenous infants hospitalized with bronchiolitis: A multicentre, randomized, placebo-controlled trial. *Front. Pediatr.* **2015**, *3*, 32.
154. McDonald, E.; Cunningham, T.; Slavin, N. Evaluating a handwashing with soap program in Australian remote Aboriginal communities: A pre and post intervention study design. *BMC Public Health* **2015**, *15*, 1188.
155. McHugh, L.; Binks, M.; Ware, R.S.; Snelling, T.; Nelson, S.; Nelson, J.; Dunbar, M.; Mulholland, E.K.; Andrews, R.M. Birth outcomes in Aboriginal mother-infant pairs from the Northern Territory, Australia, who received 23-valent polysaccharide pneumococcal vaccination during pregnancy, 2006–2011, the PneuMum randomised controlled trial. *Aust. N. Z. J. Obstet. Gynaecol.* **2019**, *60*, 82–87.
156. McKay, C.C.; Chang, A.B.; Versteegh, L.A.; McCallum, G.B. Culturally appropriate flipcharts improve the knowledge of common respiratory conditions among Northern Territory Indigenous families. *Health Promot. J. Austr.* **2015**, *26*, 150–153.
157. McMahon, E.; Webster, J.; Brimblecombe, J. Effect of 25% Sodium reduction on sales of a top-selling bread in remote Indigenous Australian community stores: A controlled intervention trial. *Nutrients* **2017**, *9*, 214.
158. McRae, M.; Taylor, S.J.; Swain, L.; Sheldrake, C. Evaluation of a pharmacist-led, medicines education program for Aboriginal Health Workers. *Rural. Remote Health* **2008**, *8*, 946.
159. Medlin, L.G.; Chang, A.B.; Fong, K.; Jackson, R.; Bishop, P.; Dent, A.; Hill, D.C.; Vincent, S.; O'Grady, K.A. Indigenous Respiratory Outreach Care: The first 18 months of a specialist respiratory outreach service to rural and remote Indigenous communities in Queensland, Australia. *Aust. Health Rev.* **2014**, *38*, 447–453.
160. Meihubers, S. The Bila Muuji oral health promotion partnership. *NSW Public Health Bull.* **2013**, *24*, 128–130.
161. Mendham, A.E.; Coutts, A.J.; Duffield, R. The acute effects of aerobic exercise and modified rugby on inflammation and glucose homeostasis within Indigenous Australians. *Eur. J. Appl. Physiol.* **2012**, *112*, 3787–3795.
162. Mendham, A.E.; Duffield, R.; Marino, F.; Coutts, A.J. Differences in post-exercise inflammatory and glucose regulatory response between sedentary indigenous Australian and Caucasian men completing a single bout of cycling. *Am. J. Hum. Biol.* **2014**, *26*, 208–214.
163. Mendham, A.E.; Duffield, R.; Marino, F.; Coutts, A.J. A 12-week sports-based exercise programme for inactive Indigenous Australian men improved clinical risk factors associated with type 2 diabetes mellitus. *J. Sci. Med. Sport* **2015**, *18*, 438–443.
164. Meyer, J.; Johnson, K.; Bowyer, J.; Muir, J.; Turner, A. Evaluating a health video on diabetic retinopathy. *Health Promot. J. Austr.* **2016**, *27*, 84–87.
165. Mills, K.; Gatton, M.L.; Mahoney, R.; Nelson, A. 'Work it out': Evaluation of a chronic condition self-management program for urban Aboriginal and Torres Strait Islander people, with or at risk of cardiovascular disease. *BMC Health Serv. Res.* **2017**, *17*, 680.

166. Morris, P.S.; Gadil, G.; McCallum, G.B.; Wilson, C.A.; Smith-Vaughan, H.C.; Torzillo, P.; Leach, A.J. Single-dose azithromycin versus seven days of amoxycillin in the treatment of acute otitis media in Aboriginal children (AATAAC): A double blind, randomised controlled trial. *Med. J. Aust.* **2010**, *192*, 24–29.
167. Munro, A.; Allan, J.; Shakeshaft, A.; Snijder, M. Riding the rural radio wave: The impact of a community-led drug and alcohol radio advertising campaign in a remote Australian Aboriginal community. *Aust. J. Rural. Health* **2017**, *25*, 290–297.
168. Nagel, T.; Robinson, G.; Condon, J.; Trauer, T. Approach to treatment of mental illness and substance dependence in remote Indigenous communities: Results of a mixed methods study. *Aust. J. Rural. Health* **2009**, *17*, 174–182.
169. Nguyen, H.; Zarnowiecki, D.; Segal, L.; Gent, D.; Silver, B.; Boffa, J. Feasibility of implementing infant home visiting in a Central Australian Aboriginal community. *Prev. Sci.* **2018**, *19*, 966–976.
170. Nguyen, K.H.; Smith, A.C.; Armfield, N.R.; Bensink, M.; Scuffham, P.A. Cost-effectiveness analysis of a mobile ear screening and surveillance service versus an outreach screening, surveillance and surgical service for Indigenous children in Australia. *PLoS One* **2015**, *10*, e0138369.
171. Noble, N.; Paul, C.; Carey, M.; Blunden, S.; Turner, N. A randomised trial assessing the acceptability and effectiveness of providing generic versus tailored feedback about health risks for a high need primary care sample. *BMC Fam. Pract.* **2015**, *16*, 95.
172. O'Donoghue, L.; Percival, N.; Laycock, A.; McCalman, J.; Tsey, K.; Armit, C.; Bailie, R. Evaluating Aboriginal and Torres Strait Islander health promotion activities using audit and feedback. *Aust. J. Prim. Health* **2014**, *20*, 339–344.
173. O'Grady, K.A.; Dunbar, M.; Medlin, L.G.; Hall, K.K.; Toombs, M.; Meiklejohn, J.; McHugh, L.; Massey, P.D.; Creighton, A.; Andrews, R.M. Uptake of influenza vaccination in pregnancy amongst Australian Aboriginal and Torres Strait Islander women: A mixed-methods pilot study. *BMC Res. Notes* **2015**, *8*, 169.
174. O'Grady, K.A.; Torzillo, P.J.; Ruben, A.R.; Taylor-Thomson, D.; Valery, P.C.; Chang, A.B. Identification of radiological alveolar pneumonia in children with high rates of hospitalized respiratory infections: Comparison of WHO-defined and pediatric pulmonologist diagnosis in the clinical context. *Pediatr. Pulmonol.* **2012**, *47*, 386–392.
175. O'Halloran, R.A.; Turner, A.W. Evaluating the impact of optical coherence tomography in diabetic retinopathy screening for an Aboriginal population. *Clin. Exp. Ophthalmol.* **2018**, *46*, 116–121.
176. Ong, K.S.; Carter, R.; Vos, T.; Kelaher, M.; Anderson, I. Cost-effectiveness of interventions to prevent cardiovascular disease in Australia's indigenous population. *Heart Lung Circ.* **2014**, *23*, 414–421.
177. Panaretto, K.S.; Gardner, K.L.; Button, S.; Carson, A.; Schibasaki, R.; Wason, G.; Baker, D.; Mein, J.; Dellit, A.; Lewis, D.; et al. Prevention and management of chronic disease in Aboriginal and Islander Community Controlled Health Services in Queensland: A quality improvement study assessing change in selected clinical performance indicators over time in a cohort of services. *BMJ Open* **2013**, *3*, e002083.
178. Passmore, E.; Shepherd, B.; Milat, A.; Maher, L.; Hennessey, K.; Havrlant, R.; Maxwell, M.; Hodge, W.; Christian, F.; Richards, J.; et al. The impact of a community-led program promoting weight loss and healthy living in Aboriginal communities: The New South Wales Knockout Health Challenge. *BMC Public Health* **2017**, *17*, 951.
179. Payne, C. A diabetes support group for Nywaigi women to enhance their capacity for maintaining physical and mental wellbeing. *Contemp. Nurse* **2013**, *46*, 41–45.
180. Peiris, D.; Wright, L.; News, M.; Corcoran, K. Community-based chronic disease prevention and management for Aboriginal people in New South Wales, Australia: Mixed methods evaluation of the 1 Deadly Step Program. *JMIR Mhealth Uhealth* **2019**, *7*, e14259.
181. Peiris, D.; Wright, L.; News, M.; Rogers, K.; Redfern, J.; Chow, C.; Thomas, D. A smartphone app to assist smoking cessation among Aboriginal Australians: Findings from a pilot randomized controlled trial. *JMIR Mhealth Uhealth* **2019**, *7*, e12745.
182. Pettigrew, S.; Jongenelis, M.I.; Moore, S.; Pratt, I.S. A comparison of the effectiveness of an adult nutrition education program for Aboriginal and non-Aboriginal Australians. *Soc. Sci. Med.* **2015**, *145*, 120–124.
183. Phillips, J.H.; Wigger, C.; Beissbarth, J.; McCallum, G.B.; Leach, A.; Morris, P.S. Can mobile phone multimedia messages and text messages improve clinic attendance for Aboriginal children with chronic otitis media? A randomised controlled trial. *J. Paediatr. Child Health* **2014**, *50*, 362–367.
184. Poder, N.; Khan, R.J.; Kovai, V.; Robinson, L.; Wright, D.; Spinks, M.; Heathcote, J.; Millen, E.; Welsh, K.; Bedford, K. Evaluating an Aboriginal tobacco social marketing project in Sydney, Australia. *Health Promot. J. Austr.* **2019**, *31*, 26–37.
185. Prowse, P.T.; Nagel, T. Developing an instrument for assessing fidelity of motivational care planning: The Aboriginal and Islander Mental health initiative adherence scale. *Int. J. Ment. Health Syst.* **2014**, *8*, 36.

186. Quilty, S.; Wood, L.; Scrimgeour, S.; Shannon, G.; Sherman, E.; Lake, B.; Budd, R.; Lawton, P.; Moloney, M. Addressing Profound disadvantages to improve Indigenous health and reduce hospitalisation: A collaborative community program in remote Northern Territory. *Int. J. Environ. Res. Public Health* **2019**, *16*, 4306.
187. Quinn, E.; O'Hara, B.J.; Ahmed, N.; Winch, S.; McGill, B.; Banovic, D.; Maxwell, M.; Rissel, C. Enhancing the get healthy information and coaching service for Aboriginal adults: Evaluation of the process and impact of the program. *Int. J. Equity Health* **2017**, *16*, 168.
188. Raphiphatthana, B.; Sweet, M.; Puszka, S.; Dingwall, K.; Nagel, T. Evaluation of a three-phase implementation program in enhancing e-mental health adoption within Indigenous primary healthcare organisations. *BMC Health Serv. Res.* **2020**, *20*, 576.
189. Read, C.; Mitchell, A.G.; de Dassel, J.L.; Scrine, C.; Hendrickx, D.; Bailie, R.S.; Johnston, V.; Maguire, G.P.; Schultz, R.; Carapetis, J.R.; et al. Qualitative evaluation of a complex intervention to improve rheumatic heart disease secondary prophylaxis. *J. Am. Heart Assoc.* **2018**, *7*, e009376.
190. Reath, J.; Carey, M. Breast and cervical cancer in indigenous women-overcoming barriers to early detection. *Aust. Fam. Physician.* **2008**, *37*, 178–182.
191. Reeve, C.; Banfield, S.; Thomas, A.; Reeve, D.; Davis, S. Community outreach midwifery-led model improves antenatal access in a disadvantaged population. *Aust. J. Rural. Health* **2016**, *24*, 200–206.
192. Reeve, C.; Humphreys, J.; Wakerman, J.; Carter, M.; Carroll, V.; Reeve, D. Strengthening primary health care: Achieving health gains in a remote region of Australia. *Med. J. Aust.* **2015**, *202*, 483–487.
193. Reeve, C.; Thomas, A.; Mossenson, A.; Reeve, D.; Davis, S. Evaluation of an ear health pathway in remote communities: Improvements in ear health access. *Aust. J. Rural. Health* **2014**, *22*, 127–132.
194. Reilly, R.E.; Cincotta, M.; Doyle, J.; Firebrace, B.R.; Cargo, M.; van den Tol, G.; Morgan-Bulled, D.; Rowley, K.G. A pilot study of Aboriginal health promotion from an ecological perspective. *BMC Public Health* **2011**, *11*, 749.
195. Ritchie, B.K.; Brewster, D.R.; Tran, C.D.; Davidson, G.P.; McNeil, Y.; Butler, R.N. Efficacy of Lactobacillus GG in aboriginal children with acute diarrhoeal disease: A randomised clinical trial. *J. Pediatr. Gastroenterol. Nutr.* **2010**, *50*, 619–624.
196. Roberts, K.; Cannon, J.; Atkinson, D.; Brown, A.; Maguire, G.; Remenyi, B.; Wheaton, G.; Geelhoed, E.; Carapetis, J.R. Echocardiographic screening for rheumatic heart disease in Indigenous Australian children: A cost-utility analysis. *J.. Am Heart Assoc.* **2017**, *6*, e004515.
197. Robertson, J.; Pointing, B.S.; Stevenson, L.; Clough, A.R. "We made the rule, we have to stick to it": Towards effective management of environmental tobacco smoke in remote Australian Aboriginal communities. *Int. J. Environ. Res. Public Health* **2013**, *10*, 4944–4966.
198. Roberts-Thomson, K.F.; Ha, D.H.; Wooley, S.; Meihubers, S.; Do, L.G. Community trial of silver fluoride treatment for deciduous dentition caries in remote Indigenous communities. *Aust. Dent. J.* **2019**, *64*, 175–180.
199. Roberts-Thomson, K.F.; Slade, G.D.; Bailie, R.S.; Endean, C.; Simmons, B.; Leach, A.J.; Raye, I.; Morris, P.S. A comprehensive approach to health promotion for the reduction of dental caries in remote Indigenous Australian children: A clustered randomised controlled trial. *Int. Dent. J.* **2010**, *60*, 245–249.
200. Robinson, G.W.; Lee, E.; Silburn, S.R.; Nagel, P.; Leckning, B.; Midford, R. School-based prevention in very remote settings: A feasibility trial of methods and measures for the evaluation of a social emotional learning program for Indigenous students in remote Northern Australia. *Front. Public Health* **2020**, *17*, e552878.
201. Schmidt, B.; Campbell, S.; McDermott, R. Community health workers as chronic care coordinators: Evaluation of an Australian Indigenous primary health care program. *Aust. N. Z. J. Public Health* **2016**, *40* (Suppl S1), S107–S114.
202. Schultz, R. Prevalences of overweight and obesity among children in remote Aboriginal communities in central Australia. *Rural. Remote Health* **2012**, *12*, 68–74.
203. Seear, K.H.; Atkinson, D.N.; Henderson-Yates, L.M.; Lelievre, M.P.; Marley, J.V. Maboo wirriya, be healthy: Community-directed development of an evidence-based diabetes prevention program for young Aboriginal people in a remote Australian town. *Eval. Program. Plann.* **2020**, *81*, e101818.
204. Segal, L.; Nguyen, H.; Schmidt, B.; Wenitong, M.; McDermott, R.A. Economic evaluation of Indigenous health worker management of poorly controlled type 2 diabetes in north Queensland. *Med. J. Aust.* **2016**, *204*, 196.
205. Shephard, M.; O'Brien, C.; Burgoyne, A.; Croft, J.; Garlett, T.; Barancek, K.; Halls, H.; McAteer, B.; Motta, L.; Shephard, A. Review of the cultural safety of a national Indigenous point-of-care testing program for diabetes management. *Aust. J. Prim. Health* **2016**, *22*, 368–374.
206. Shephard, M.; Shephard, A.; McAteer, B.; Regnier, T.; Barancek, K. Results from 15years of quality surveillance for a National Indigenous Point-of-Care Testing Program for diabetes. *Clin. Biochem.* **2017**, *50*, 1159–1163.

207. Shield, J.M.; Kearns, T.M.; Garenggulkpuy, J.; Walpulay, L.; Gundjirryirr, R.; Bundhala, L.; Djarpanbuluwuy, V.; Andrews, R.M.; Judd, J. Cross-cultural, Aboriginal language, discovery education for health literacy and informed consent in a remote Aboriginal community in the Northern Territory, Australia. *Trop. Med. Infect. Dis.* **2018**, *3*, 15.
208. Sinclair, C.; Stokes, A.; Jeffries-Stokes, C.; Daly, J. Positive community responses to an arts-health program designed to tackle diabetes and kidney disease in remote Aboriginal communities in Australia: A qualitative study. *Aust. N. Z. J. Public Health* **2016**, *40*, 307–312.
209. Slade, G.D.; Bailie, R.S.; Roberts-Thomson, K.; Leach, A.J.; Raye, I.; Endean, C.; Simmons, B.; Morris, P. Effect of health promotion and fluoride varnish on dental caries among Australian Aboriginal children: Results from a community-randomized controlled trial. *Community Dent. Oral. Epidemiol.* **2011**, *39*, 29–43.
210. Smith, A.C.; Armfield, N.R.; Wu, W.I.; Brown, C.A.; Mickan, B.; Perry, C. Changes in paediatric hospital ENT service utilisation following the implementation of a mobile, indigenous health screening service. *J. Telemed. Telecare* **2013**, *19*, 397–400.
211. Smith, A.C.; Armfield, N.R.; Wu, W.; Brown, C.A.; Perry, C. A mobile telemedicine-enabled ear screening service for Indigenous children in Queensland: Activity and outcomes in the first three years. *J. Telemed. Telecare* **2012**, *18*, 485–489.
212. Smith, A.C.; Brown, C.; Bradford, N.; Caffery, L.J.; Perry, C.; Armfield, N.R. Monitoring ear health through a telemedicine-supported health screening service in Queensland. *J. Telemed. Telecare* **2015**, *21*, 427–430.
213. Smith, L.; Blinkhorn, F.; Moir, R.; Blinkhorn, A. Results of a two year dental health education program to reduce dental caries in young Aboriginal children in New South Wales, Australia. *Community Dent. Health* **2018**, *35*, 211–216.
214. Smithers, L.G.; Lynch, J.; Hedges, J.; Jamieson, L.M. Diet and anthropometry at 2 years of age following an oral health promotion programme for Australian Aboriginal children and their carers: A randomised controlled trial. *Br. J. Nutr.* **2017**, *118*, 1061–1069.
215. Snodgrass, W.J.; Rayner, V.; Rice, S.M.; Purcell, R.; Bowers, J. Evaluation of a culturally sensitive social and emotional well-being program for Aboriginal and Torres Strait Islanders. *Aust. J. Rural. Health* **2020**, *28*, 327–337.
216. Soares, G.H.; Santiago, P.H.R.; Biazevic, M.G.H.; Michel-Crosato, E.; Jamieson, L. Do network centrality measures predict dental outcomes of Indigenous children over time? *Int. J. Paediatr. Dent.* **2020**, *31*, 634–646.
217. Spaeth, B.A.; Shephard, M.D.; Schatz, S. Point-of-care testing for haemoglobin A1c in remote Australian Indigenous communities improves timeliness of diabetes care. *Rural. Remote Health* **2014**, *14*, 136–140.
218. Spurling, G.K.; Askew, D.A.; Schluter, P.J.; Hayman, N.E. Implementing computerised Aboriginal and Torres Strait Islander health checks in primary care for clinical care and research: A process evaluation. *BMC Med. Inform. Decis. Mak.* **2013**, *13*, 108.
219. Spurling, G.K.; Hayman, N.E.; Cooney, A.L. Adult health checks for Indigenous Australians: The first years' experience from the Inala Indigenous Health Service. *Med. J. Aust.* **2009**, *190*, 562–564.
220. Standen, J.C.; Morgan, G.G.; Sowerbutts, T.; Blazek, K.; Gugusheff, J.; Puntsag, O.; Wollan, M.; Torzillo, P. Prioritising housing maintenance to improve health in Indigenous communities in NSW over 20 years. *Int. J. Environ. Res. Public Health* **2020**, *17*, 5946.
221. Stanley, S.H.; Laugharne, J.; Chapman, M.; Balaratnasingam, S. The physical health of Indigenous people with a mental illness in the Kimberley: Is ongoing monitoring effective? *Australas Psychiatr.* **2019**, *27*, 358–361.
222. Stephen, A.T.; Leach, A.J.; Morris, P.S. Impact of swimming on chronic suppurative otitis media in Aboriginal children: A randomised controlled trial. *Med. J. Aust.* **2013**, *199*, 51–55.
223. Sun, J.; Buys, N. Effects of community singing program on mental health outcomes of Australian Aboriginal and Torres Strait Islander people: A meditative approach. *Am. J. Health Promot.* **2016**, *30*, 259–263.
224. Tan, J.K.; Kearns, P.; Martin, A.C.; Siafarikas, A. Randomised controlled trial of daily versus stoss vitamin D therapy in Aboriginal children. *J. Paediatr. Child Health* **2015**, *51*, 626–631.
225. Tane, M.P.; Hefler, M.; Thomas, D.P. An evaluation of the 'Yaka ENGarali" Tackling Indigenous Smoking program in East Arnhem Land: Yol engu people and their connection to engarali'. *Health Promot. J. Austr.* **2018**, *29*, 10–17.
226. Thomas, D.P.; McMahon, E.; Wang, Z.; Scollo, M.M.; Durkin, S.J. Impact of three annual tobacco tax rises on tobacco sales in remote Australian Aboriginal community stores. *Tob. Control.* **2020**, *30*, e122–e127.
227. Thomas, D.; Johnston, V.; Fitz, J. Lessons for Aboriginal tobacco control in remote communities: An evaluation of the Northern Territory 'Tobacco Project'. *Aust. N. Z. J. Public Health* **2010**, *34*, 45–49.
228. Thomas, P.; Joseph, T.L.; Menzies, R.I. Evaluation of a targeted immunisation program for Aboriginal and Torres Strait Islander infants in an urban setting. *NSW Public Health Bull.* **2008**, *19*, 96–99.

229. Thornton, R.B.; Kirkham, L.S.; Corscadden, K.J.; Coates, H.L.; Vijayasekaran, S.; Hillwood, J.; Toster, S.; Edminston, P.; Zhang, G.; Keil, A.; et al. No evidence for impaired humoral immunity to pneumococcal proteins in Australian Aboriginal children with otitis media. *Int. J. Pediatr. Otorhinolaryngol.* **2017**, *92*, 119–125.
230. Thornton, R.B.; Kirkham, L.S.; Corscadden, K.J.; Wiertsema, S.P.; Fuery, A.; Jones, B.J.; Coates, H.L.; Vijayasekaran, S.; Zhang, G.; Keil, A.; et al. Australian Aboriginal Children with otitis media have reduced antibody titers to specific nontypeable haemophilus influenzae vaccine antigens. *Clin. Vaccine Immunol.* **2017**, *24*, e00556-16.
231. Tighe, J.; Shand, F.; Ridani, R.; Mackinnon, A.; De La Mata, N.; Christensen, H. Ibobbly mobile health intervention for suicide prevention in Australian Indigenous youth: A pilot randomised controlled trial. *BMJ Open* **2017**, *7*, e013518.
232. Tong, S.Y.; Andrews, R.M.; Kearns, T.; Gundjirryirr, R.; McDonald, M.I.; Currie, B.J.; Carapetis, J.R. Trimethopim-sulfamethoxazole compared with benzathine penicillin for treatment of impetigo in Aboriginal children: A pilot randomised controlled trial. *J. Paediatr. Child Health* **2010**, *46*, 131–133.
233. Treloar, C.; Hopwood, M.; Cama, E.; Saunders, V.; Jackson, L.C.; Walker, M.; Ooi, C.; Ubrihien, A.; Ward, J. Evaluation of the Deadly Liver Mob program: Insights for roll-out and scale-up of a pilot program to engage Aboriginal Australians in hepatitis C and sexual health education, screening, and care. *Harm. Reduct. J.* **2018**, *15*, 5.
234. Valery, P.C.; Masters, I.B.; Taylor, B.; Laifoo, Y.; O'Rourke, P.K.; Chang, A.B. An education intervention for childhood asthma by Aboriginal and Torres Strait Islander health workers: A randomised controlled trial. *Med. J. Aust.* **2010**, *192*, 574–579.
235. Vallesi, S.; Wood, L.; Dimer, L.; Zada, M. "In Their Own Voice"—Incorporating underlying social determinants into Aboriginal health promotion programs. *Int. J. Environ. Res. Public Health* **2018**, *15*, 1514.
236. Ward, J.S.; Dyda, A.; McGregor, S.; Rumbold, A.; Garton, L.; Donovan, B.; Kaldor, J.M.; Guy, R.J. Low HIV testing rates among people with a sexually transmissible infection diagnosis in remote Aboriginal communities. *Med. J. Aust.* **2016**, *205*, 168–171.
237. Ward, J.S.; Guy, R.J.; Akre, S.P.; Wand, H.; McManus, H.; Dyda, A.; Garton, L.; Hengel, B.; Silver, B.J.; Taylor-Thomson, D.; et al. Epidemiology of syphilis in Australia: Moving toward elimination of infectious syphilis from remote Aboriginal and Torres Strait Islander communities? *Med. J. Aust.* **2011**, *194*, 525–529.
238. Whiteside, M.; Tsey, K.; Crouch, A.; Fagan, P. Youth and Relationship Networks (YARNS): Mobilising communities for sexual health. *Health Promot. J. Austr.* **2012**, *23*, 226–230.
239. Xu, H.; Geros, C.; Turner, E.; Egan, M.; Cocotis, K.; Mitchell, C.; Arambasic, N.; Browne, J. Feltman: Evaluating the utilisation of an Aboriginal diabetes education tool by health professionals. *Aust. J. Prim. Health* **2018**, *24*, 496–501.
240. Young, C.; Gunasekera, H.; Kong, K.; Purcell, A.; Muthayya, S.; Vincent, F.; Wright, D.; Gordon, R.; Bell, J.; Gillor, G.; et al. A case study of enhanced clinical care enabled by Aboriginal health research: The Hearing, EAr health and Language Services (HEALS) project. *Aust. N. Z. J. Public Health* **2016**, *40*, 523–528.
